# One- and two-hour storage delays improve nutritional components in *Sarcocornia fruticosa* ecotypes, but not in the *Arthrocaulon macrostachyum* ecotype

**DOI:** 10.3389/fpls.2026.1943035

**Published:** 2026-10-06

**Authors:** Tesfaye Asmare Sisay, Jaykumar Patel, Kusum Khatri, Babita Choudhary, Dominic Standing, Zai Du Nja, Itai Kolsky, Muki Shpigel, Luísa Margarida Batista Custódio, Ilya Gelfand, Moshe Sagi

**Affiliations:** 1 The Albert Katz International School for Desert Studies, The Jacob Blaustein Institutes for Desert Research, Ben-Gurion University of the Negev, Sede Boker Campus, Midreshet Ben-Gurion, Israel; 2 Jacob Blaustein Center for Scientific Cooperation, The Jacob Blaustein Institutes for Desert Research, Ben-Gurion University of the Negev, Sede Boker Campus, Midreshet Ben-Gurion, Israel; 3 The Albert Katz Department of Dryland Biotechnologies, French Associates Institute for Agriculture and Biotechnology of Dryland, The Jacob Blaustein Institutes for Desert Research, Ben-Gurion University of the Negev, Sede Boker Campus, Midreshet Ben-Gurion, Israel; 4 Morris Kahn Marine Research Station, The Leon H. Charney School of Marine Sciences, University of Haifa, Haifa, Israel; 5 Center of Marine Sciences, University of Algarve, Campus de Gambelas, Faro, Portugal; 6 Katif Research Center, Sedot Negev, Israel; 7 Ministry of Science and Technology, Netivot, Israel

**Keywords:** antioxidant, *Arthrocaulon macrostachyum*, halophytes, postharvest management, storage delay, water loss

## Abstract

The effects of postharvest storage delays of 1 and 2 hours on the nutritional quality of halophytic plants irrigated with 100 and 150 mM NaCl solutions were assessed. The study compared the new *Sarcocornia fruticosa* ecotypes Shikmona, Megadim, and Ruhama with the commonly cultivated VM and *Arthrocaulon macrostachyum* (AM). All new ecotypes outperformed the VM ecotype in terms of nutritional composition and yield. Except for Shikmona, the biomass of all ecotypes increased with elevated salinity; however, storage delays reduced water content, with a greater reduction under lower salinity. Storage delays in *Sarcocornia* significantly enhanced total polyphenols, flavonoids, carotenoids, anthocyanins, and radical-scavenging activity compared with levels in the plant contents immediately after harvesting. In contrast, AM showed declines in antioxidants, except for anthocyanins and carotenoids, after 1- and 2-hour delays. Both storage delays increased total soluble sugars, electrical conductivity, and malondialdehyde in AM and the *Sarcocornia* ecotypes. Further carotenoid enhancement in AM was observed in the 2-hour storage-delay treatment, followed by 21 days of storage, compared with the control stored immediately for 21 days. Across *Sarcocornia* ecotypes, storage delay is associated with increased levels of polyphenols, flavonoids, and carotenoids, as well as enhanced radical-scavenging activity. In contrast, AM showed decreased total flavonoids, polyphenols, and radical scavenging activity after a 2-hour storage delay, followed by 21 days of cold room storage, compared to 21 days of storage without a delay. Yet, its antioxidant content remained higher than that of the *Sarcocornia* ecotypes in both the non-delayed control and the 2-hour storage-delay treatments.

## Highlights

- Irrigation with a higher salinity solution reduced water loss during postharvest storage delays.
- Storage delays of *S. fruticosa* ecotypes for one to two hours enhanced their antioxidant capacity, while decreasing the antioxidant capacity of *Arthrocaulon macrostachyum*.
- Storage delays increased antioxidant activity, radical-scavenging activity, and malondialdehyde levels in *Sarcocornia* ecotypes beyond the effects of water loss.
- Storage delays decreased antioxidant-related parameters and radical-scavenging activity in *Arthrocaulon macrostachyum* beyond the effects of water loss.
- A two-hour postharvest delay followed by 21 days of cold storage enhanced antioxidant capacity in *S. fruticosa* ecotypes but reduced it in *Arthrocaulon macrostachyum*.

## 1 Introduction

Humans have long used halophyte plants as natural salt substitutes in food (Antunes et al., 2021; Ventura et al., 2015) and as traditional medicinal plants (Ventura et al., 2014). The halophytic plants *Sarcocornia* and *Salicornia*, commonly sold as “samphire” or “sea asparagus,” are highly valued in European and Asian markets as fresh vegetables and garnishes in gourmet food (Antunes et al., 2021; Ventura et al., 2011a). *Arthrocaulon macrostachyum* and *Sarcocornia fruticosa* genotypes are excellent sources of nutrients and antioxidants (Barreira et al., 2017). *Sarcocornia* ecotypes, as perennials, can be harvested multiple times throughout the year (Sisay et al., 2025; Ventura et al., 2011b).

Various polyphenols, common phytochemicals with antioxidant activity, are abundant in *Sarcocornia* and related halophytes, garnering the attention of the food and pharmaceutical industries (Fitzner et al., 2023; Maisuthisakul et al., 2008). Such antioxidants can protect against oxidative stress damage, reduce the risk of chronic diseases, and eliminate potentially harmful free radicals in the human body (Barreira et al., 2017; Sisay et al., 2025).

Postharvest management strategies are a crucial aspect of agricultural activity, particularly for high-water-content fresh vegetables and fruits, helping extend their shelf life and maintain product quality (Kaur and Kaur, 2022; Valenzuela, 2023). Horticultural products typically contain high moisture levels and, as such, may often suffer quality and quantity loss between harvest and consumption (Adhikari and G.C., 2021; Kader, 2005). Water loss after harvest is a primary cause of quality degradation, leading to wilting, shriveling, and softening that may affect the marketability of crop products, depending on the time elapsed between harvest and storage (Amit et al., 2017; Gidado et al., 2024). However, in certain situations, storage delays can enhance nutrient and flavor profiles, minimize spoilage, and enable bulk storage for extended periods (Amit et al., 2017).

Numerous studies have demonstrated substantial degradation of bioactive compounds in vegetable crops during cold storage. For instance, Galani et al. (2017) reported significant reductions in DPPH radical-scavenging activity, vitamin C, and total polyphenol content in leafy vegetables such as amaranth, dill, onion leaves, fenugreek, and spinach after 15 days of storage at 4 °C. Similarly, Gil et al. (1999) observed pronounced decreases in free radical-scavenging capacity, total flavonoids, and vitamin C in spinach stored at 10 °C for seven days. In edible halophytes, Gago et al. (2011) reported marked deterioration in the quality of *Sarcocornia perennis* and *Salicornia ramosissima* during 21 days of storage at 1, 4, and 9 °C. This deterioration included visible discoloration from green to brown, reflecting progressive postharvest quality loss. While previous research has mostly examined how refrigeration storage at different temperatures and durations affects the quality of vegetables and some edible halophytes, the impact of the delay in storage time between harvest and refrigeration storage has not been thoroughly investigated. Furthermore, it is unclear if preharvest salinity influences how edible halophytes physiologically, biochemically, and nutritionally respond to postharvest storage delays.

The current study provides a comprehensive evaluation of the interactive effects of preharvest salinity level treatment and postharvest storage delays before refrigeration on the physiological, biochemical responses, and nutritional quality of edible halophytes, which have not been studied before.

The specific objectives of this study were: (i) To determine the effects of one- and two-hour postharvest delays before cold storage on the physiological, biochemical, and nutritional quality of selected *Sarcocornia* ecotypes and *Arthrocaulon macrostachyum*. (ii) To evaluate whether plants grown under different salinity levels (100 and 150 mM NaCl) respond differently to one- and two-hour postharvest storage delays. (iii) To assess the impact of a two-hour postharvest delay, followed by 21 days of cold storage, on quality retention. To achieve these objectives, we analyzed key quality indicators, including total phenolic and flavonoid content, photosynthetic pigments (carotenoids and anthocyanins), antioxidant radical-scavenging activity, and oxidative stress markers. This study is expected to advance our understanding of how preharvest and postharvest factors interact to affect the nutritional quality and antioxidant quality of edible halophytes.

Delaying storage in *Sarcocornia* by one or two hours significantly enhanced the total polyphenols, flavonoids, carotenoids, anthocyanins, and radical scavenging activity, compared with the immediately stored control. This enhancement in the storage-delay samples was also observed after storing the control and the two-hour storage-delay samples for 21 days. Notably, the new *Sarcocornia* ecotypes, Shikmona (Shik), Megadim (Meg), and Ruhama (Ruh), outperformed the locally used VM cultivar in both nutritional composition and yield. Furthermore, the one- and two-hour storage delays significantly increased the *Sarcocornia* shoot product’s antioxidant capacity beyond the expected increase due to water loss. In contrast, AM exhibited a decrease in antioxidant levels, except for total anthocyanins and carotenoids. Both storage delay times increased total soluble sugar, EC, and malondialdehyde levels in the AM and *Sarcocornia* ecotypes. These findings provide a scientific foundation for optimizing harvest and postharvest management strategies, thereby improving quality retention, extending shelf life, and enhancing the agronomic and commercial potential of halophytes grown in saline conditions.

## 2 Materials and methods

### 2.1 Experimental setup

The study used the *Sarcocornia* ecotypes VM, Shikmona (Shik), Megadim (Meg), and Ruhama (Ruh), together with *Arthrocaulon macrostachyum* ecotype. The nomenclature and taxonomic identification of the different ecotypes follow the updated classification framework and have been previously reported. Detailed methodological information is provided in the Materials and Methods section, and the supporting sequence data and phylogenetic analyses are included in the **Supplementary Materials** (Sisay et al., 2025).

Germination and growth conditions were the same as those used previously (Sisay et al., 2022, 2025). In brief, seeds were germinated in non-saline tap water and acclimated to low salinity (1 g/L NaCl) water. Once they reached approximately 2 cm in height, the seedlings were transplanted into 3-L pots containing autoclaved potting soil and were irrigated with a 50-mM NaCl solution supplemented with 1 g/L of NPK fertilizer + micronutrients (20-20-20, Haifa Chemicals, Haifa, Israel). The nitrogen molecules in the 20-20–20 were ammonium (1.5 mM), nitrate (2.2 mM), and urea (3.7 mM). K and P were 0.3 mM P as P_2_O_5_ and 0.4 mM K as K_2_O. Micronutrients were Mo 70 ppm, Cu 110 ppm, Zn 150 ppm, Mn 500 ppm, and Fe 1000 ppm. This regime continued until the start of the formal experiments. After three months of growth, the plants were given a technical cutting, removing shoot material 10 cm above soil level (referred to as the “cutting table”) to standardize growth before the main experimental phase. After this, plants were grown under one of two salinity treatments: either 100 mM or 150 mM NaCl, including 1 g L^−1^ NPK nutrients [20-20-20 + micronutrients (Haifa Chemicals, Israel)]. In addition, the nutrient solution was supplemented with 4 mM of NH_4_NO_3_.

Each pot contained 14 uniformly sized plants, with 8 replicate pots per treatment (5 ecotypes × 2 salinity treatments × 8 replicates). The plants were cultivated in a semi-controlled greenhouse under natural day lengths and with a temperature range of 15 °C to 40 °C, a relative humidity of 75%, and a PAR (photosynthetically active radiation) of 650 to 700 µmol m^-2^ s^−1^ throughout the experiments. Harvesting took place every 30 days, with regrown shoot material above the cutting table height collected for six harvests.

### 2.2 Harvesting regime and experimental design

The initial harvest was performed as a technical cut, removing material 10 cm above soil level to create a cutting table once plants reached 13–17 cm in height. Within 30 days of this cutting, plants regrew new shoots above the cutting table, marking the start of the first formal harvest. Fresh shoot biomass was recorded immediately after each successive 30-day harvest. Part of the harvested fresh shoot biomass was dried in a forced-air oven at 65 °C until a constant dry weight was achieved. Both fresh and dry biomass were expressed as kg m^−2^ because this unit represents biomass productivity per unit cultivation area, allowing direct comparison of yield performance among treatments and ecotypes. Expressing dry weight on an area basis also provides a meaningful estimate of dry matter production at the cultivation scale and enables conversion to an area basis, which is commonly used for evaluating agricultural productivity. Control fresh shoot samples, which included 10 shoots measuring 7 cm in length and weighing approximately 3 g, were flash-frozen in liquid nitrogen immediately after harvesting.

For the post-harvest delay study, freshly harvested shoots were weighed, and independent samples were subjected to either a 1- or 2-hour delay in a semi-controlled greenhouse under natural daylight. Both the 1- and 2-hour treatments were conducted during the same experimental period and under comparable ambient conditions, including approximately 25 °C, 75% relative humidity, and photosynthetically active radiation (PAR) of 600–650 µmol m^−2^ s^−1^. Immediately after the designated delay period, samples were reweighed, flash-frozen in liquid nitrogen, and stored at −80 °C until biochemical analysis. Thus, the primary experimental difference between these treatments was the duration of post-harvest exposure.

A separate set of independent samples was used for the 21-day cold-storage experiment. For the delayed-storage treatment, freshly harvested shoots were subjected to a 2-hour post-harvest delay under the same greenhouse conditions described above. After the delay, samples were weighed, placed in 180 cm^3^ plastic cups, covered with cling film, and transferred to a cold-storage room maintained at 4 °C in complete darkness for 21 days. At the end of the storage period, samples were weighed, immediately flash-frozen in liquid nitrogen, and stored at −80 °C until biochemical analysis.

For the corresponding cold-storage control, freshly harvested shoot samples were weighed, immediately placed in 180 cm^3^ plastic cups, covered with cling film, and transferred directly to the same cold-storage conditions (4 °C, complete darkness) for 21 days, without a pre-storage delay. Following storage, the samples were weighed, flash-frozen in liquid nitrogen, and stored at −80 °C until analysis.

Accordingly, the 1- and 2-hour delay treatments represented independent post-harvest exposure experiments. In contrast, the 21-day storage experiment independently compared samples subjected to a 2-hour pre-storage delay with samples transferred directly to cold storage. This experimental arrangement allowed the effect of post-harvest delay to be evaluated separately from that of 21-day cold storage while maintaining identical cold-storage conditions for both the delayed and non-delayed treatments.

A schematic representation (**Supplementary Figure 4**) was generated to provide a comprehensive overview of the experimental workflow, from harvesting and sample collection to the various postharvest treatments and subsequent biochemical analyses. Specifically, it illustrates the control, 1-h, and 2-h delayed-processing treatments; the 2-h delay followed by 21 days of cold storage at 4 °C; and the subsequent flash-freezing in liquid nitrogen and storage at −80 °C before analysis.

### 2.3 Water loss determination

Water loss was assessed by employing one- and two-hour postharvest delays, with 10 biological replicates per ecotype, and by comparing the weight to that recorded immediately after harvest. Samples stored in a cold room for 21 days were weighed just before storage and immediately after storage.

Water loss (%) was calculated as follows:

[(FWt_0_ − FWt_x_)/FWt_0_] × 100%. FWt_0_ represents fresh weights at harvest, and FWt_x_ represents fresh weight after one hour, after two hours, or after 21 days of storage.

### 2.4 Assessment of total soluble sugar and electrical conductivity

Sample extraction was performed as described before (Sisay et al., 2022), with slight modifications. Briefly, 200 mg of fresh tissue was ground in liquid nitrogen and mixed with deionized distilled water (1:10 w/v). Supernatants were collected following centrifugation at 18,400 rcf for 20 min at 4 °C. Electrical conductivity (EC) and total soluble solids were determined by using a standard EC meter (ECTestr 11, Eutech Instruments, a ThermoFisher Scientific manufacturing company, Paisley, UK) and a refractometer (Atago Digital Refractometer PR-1, Tokyo, Japan), respectively. Total soluble sugar and EC are expressed in % and deci-Siemens per meter (dS m^−1^), respectively.

### 2.5 Total carotenoid content determination

Total carotenoid contents were measured following the protocol of Trentin et al. (2024), with minor modifications. Fresh tissue (100 mg) was extracted with 80% ethanol (m/v, 1:10) and incubated in the dark at 4 °C for 48 h until it was colorless. The samples were centrifuged at 18,400 rcf for 15 min at 4 °C, then transferred to new tubes and centrifuged again. Next, 0.2-mL samples were taken to measure absorbance at 665 nm, 649 nm, 652 nm, and 470 nm for chlorophyll a, chlorophyll b, total chlorophyll, and total carotenoids, respectively, using a spectrophotometer (EPOCH, Agilent (BioTek), Santa Clara, CA, USA). The calculations were carried out as previously described (Ben-Amotz et al., 1988; Lichtenthaler, 1987).

1. Chlorophyll A = [(12.21 × A665) − (2.81 × A649)/w] × (V/1000)
2. Chlorophyll B = [(20.13 × A649) − (2.03 × A665)/w] × (V/1000)
3. Total carotenoid (µg g^−1^ FW) = [(1000 × Car) − (2.05 × Chl_a_) − (114 × Chl_b_)]/245

### 2.6 Anthocyanin determination

Anthocyanin concentrations were determined as described by Sisay et al. (2022), with a slight modification. Frozen fresh materials (100 mg) were first crushed in liquid nitrogen, and 5 mL of 1% HCl-acidified methanol [m/v: 1:7.5 (48.44 mL methanol plus 1.56 mL 32N HCL)] was added; the mixture was then centrifuged at 18,400 rcf for 20 min at 4 °C. Next, 500 µL of double distilled water (DDW) was added to 500 µL of the supernatant and 1 mL of chloroform. The mixture was thoroughly vortexed and then centrifuged for 20 min at 4 °C at 18,400 rcf. The absorbance of the supernatant was measured at 530 nm and 657 nm. The anthocyanin contents were calculated using the following equation:


Anthocyanin(mg/g FW)=(A530−(0.25×A657))×extraction volume(mL)×1/weight tissue(g) fresh weight.

### 2.7 Malondialdehyde determination

MDA content was estimated using the colorimetric method (Hodges et al., 1999). Samples were extracted in a 1:10 (w/v) ratio of chilled extraction buffer [1% trichloroacetic acid (TCA), 0.1 M phenylmethylsulfonyl fluoride (PMSF) in a phosphate-buffered saline solution (pH 7.4)]. Plant extracts were divided into two sets and mixed with equal amounts of 10% TCA and 0.8% thiobarbituric acid (TBA) in 10% TCA, respectively. The extraction buffer was replaced with plant extract to create a blank reaction. Tubes were heated to 99 °C for 1 h, and the absorbance of the developed color was measured at 440, 532, and 600 nm. A reaction containing 10% TCA was included as an additional control to minimize potential interference from accumulated anthocyanins, soluble sugars, and other non-specific compounds. The resulting measurements were considered TBARS-associated indicators of lipid peroxidation/oxidative stress, rather than unequivocal measurements of MDA alone. To account for potential matrix effects, TBARS values were compared among treatments under identical experimental conditions, while recognizing that treatment-related increases in carbohydrates and anthocyanins may contribute to the measured TBARS signal. MDA is expressed in nanomoles per gram fresh weight (nmol g^−1^ FW).

1. [(Abs532_+TBA_ − Abs600_+TBA_) − (Abs532_−TBA_ − Abs600_−TBA_)] = A
2. [(Abs440_+TBA_ − Abs600_+TBA_) × 0.0571] = B

MDA (nmol g^−1^ FW) = ((A − B)/157,000) × 10^6^

### 2.8 Total polyphenol content

The total polyphenol content was determined using the method described by Khatri and Rathore (2022) with slight modifications. Briefly, frozen fresh tissue (100 mg) was crushed, and 80% ice-cold methanol (m/v: 1:10) was added. The mixture was centrifuged at 22,500 rcf for 20 min at 4 °C. Then, 0.2 mL of the supernatant was mixed with 0.5 mL of Folin–Ciocalteu phenol reagent and incubated at room temperature for 5 min, then mixed with 2 mL of 20% sodium carbonate. The mixture was incubated in a heated bath at 100 °C for 5 min. Absorbance was measured at 650 nm. The standard curve was prepared using catechol concentrations from 0 to 1000 µg mL^−1^, and the total polyphenol content is reported as milligrams of catechol equivalents (CE) per gram fresh weight (mg CE g^−1^ FW).

### 2.9 Total flavonoids

The total flavonoid content was determined using a modified method previously described (Patel et al., 2022). After using liquid nitrogen to crush 100 mg of fresh, frozen material, 80% ice-cold methanol (v/v, 1:10) was added, and the mixture was centrifuged for 20 min at 4 °C at 22,500 rcf. Then, 0.1 mL of the supernatant was mixed with 0.3 mL of 5% sodium nitrate, vortexed, and incubated for 5 min at room temperature. Subsequently, 0.3 mL of aluminum chloride (10% w/v) and 2 mL of sodium hydroxide (1 M) were added, and the solution was mixed. The absorbance was recorded at 510 nm. The standard curve was prepared using quercetin at concentrations ranging from 0 to 500 µg/mL. The results are expressed as quercetin equivalents per gram of fresh weight (mg QE g^−1^ FW).

### 2.10 Radical scavenging assay

The radical scavenging activity (antioxidant activity) of the shoot extract was determined using 2,2-diphenyl-1-picrylhydrazyl (DPPH) as the free radical, as previously described (Choudhary et al., 2023), with minor modifications. The DPPH stock solution [0.024% (m/v)] was prepared in methanol, and a working solution was made by diluting the stock solution with methanol until the absorbance reached 0.98 ± 0.02 at 517 nm. The scavenging activity of the plant shoot extract was determined by adding 2 mL of DPPH solution to 100 µL of plant extract (as extracted for total polyphenol content determination). The mixture was incubated for 30 min in the dark at room temperature. The absorbance was measured at 517 nm, and Trolox was used as a standard antioxidant at concentrations ranging from 0 to 100 µM. The percentage of the samples’ DPPH radical scavenging activity was determined using the following formula:

Free radical scavenging activity(%)


$$DPPH=\;\frac{Absorbance_{517\;nm\left(control\right)}-\;Absorbance_{517\;nm\left(sample\right)}}{Absorbance_{517\;nm\left(control\right)}}\times 100$$

### 2.11 Estimating the expected results of total soluble solids, electrical conductivity, total polyphenol content, radical scavenging activity, and MDA, considering the water loss during storage delay

To differentiate between concentration changes due to water loss and those resulting from physiological or biochemical responses during postharvest delays, we calculated the expected values of EC, total soluble sugar, TPC, radical scavenging activity, and MDA for samples subjected to one-hour and two-hour storage delays. The percentage of water loss was determined by comparing the reduction in the samples’ fresh weight with that of the control samples analyzed immediately after harvest. Expected concentrations were derived by adjusting the control values based on measured water loss, assuming no metabolic changes and that any observed increases in concentration were solely due to tissue dehydration. We then compared the experimentally measured values in each delay period with the corresponding expected values to determine whether the changes were exclusively due to water loss or were influenced by additional physiological or biochemical processes induced by storage or storage delays.

### 2.12 Data analysis

Representative data for one- and two-hour delays, as well as for a two-hour delay followed by 21 days of storage in a cold room, are presented from independent experiments that yielded similar results. To account for increases in concentration due to water loss, the levels of EC, total soluble sugar, total polyphenol content, radical scavenging activity, and MDA were determined by considering the water loss during storage delays of one and two hours, compared to the actual concentration, based on the water loss percentages relative to the control plant material.

Significant differences between treatments were determined using a one-way ANOVA followed by the Tukey-Kramer HSD test at a 5% significance level [JMP8 (SAS, Cary, NC, USA)] and Student’s t-test (P< 0.05), n = 3 to 10, depending on the availability of sample materials.

Significantly, complete results of a factorial ANOVA, including all main effects and interactions, are presented in **Supplementary Table 2** and experimental layout in **Supplementary Table 3** for readers seeking additional statistical details. The factorial multi-way ANOVA was conducted to assess the main effects and interactions of ecotype, salinity level, and postharvest storage/delay treatment. The experiment employed a three-factor factorial design within a completely randomized design, rather than a split-plot design. This approach was chosen because the 0-, 1-, and 2-hour postharvest treatments were applied to independent samples within each ecotype x salinity combination, rather than being assigned as subplots within a common salinity main plot. Consequently, the experiment lacked the hierarchical structure required for a split-plot design, where salinity level would be the main factor and storage delay the split factor.

The experiment included five ecotypes (VM, Shikmona, Megadim, Ruhama, and *Arthrocaulon macrostachyum* [AM]), two salinity levels (100 and 150 mM NaCl), and three postharvest delay treatments (0 h/control, 1 h, and 2 h). Independent biological replicates were used for each treatment combination, resulting in an experimental structure of 5 ecotypes × 2 salinity levels × 3 postharvest treatments × independent replicates = experimental units.

Accordingly, the analysis utilized a factorial completely randomized design, with the residual experimental error serving as the denominator for the F-tests of the main effects and interaction terms. This eliminated the need for separate main-plot and subplot error terms. The statistical model incorporated the main effects of ecotype, salinity, and postharvest delay, along with all two-way and three-way interactions.

A principal component analysis (PCA) was conducted in JMP8 using a multivariate approach based on the correlation matrix with Varimax rotation. Two principal components were extracted, and biplots were used to visualize the relationships between water loss, malondialdehyde (MDA), total soluble sugar, electrical conductivity (EC), and antioxidant parameters across the studied *Sarcocornia fruticosa* ecotypes and the *Arthrocaulon macrostachyum* ecotype.

## 3 Results

### 3.1 Fresh and dry shoot biomass

The effects of salinity on fresh and dry shoot biomass were evaluated across six successive harvests every 30 days. Except for the Shik ecotype, the *Sarcocornia* ecotypes VM, Meg, and Ruh, as well as *Arthrocaulon macrostachyum* (AM), produced higher fresh biomass at the higher salinity level (150 mM NaCl) than at 100 mM NaCl. Under higher salinity, the Ruh ecotype accumulated the highest fresh biomass, and the Shik and VM ecotypes the lowest among the *Sarcocornia* ecotypes. However, the fresh biomass of Ruh remained lower than that of AM at both salinity levels (**Figure 1A**).

**Figure 1:**
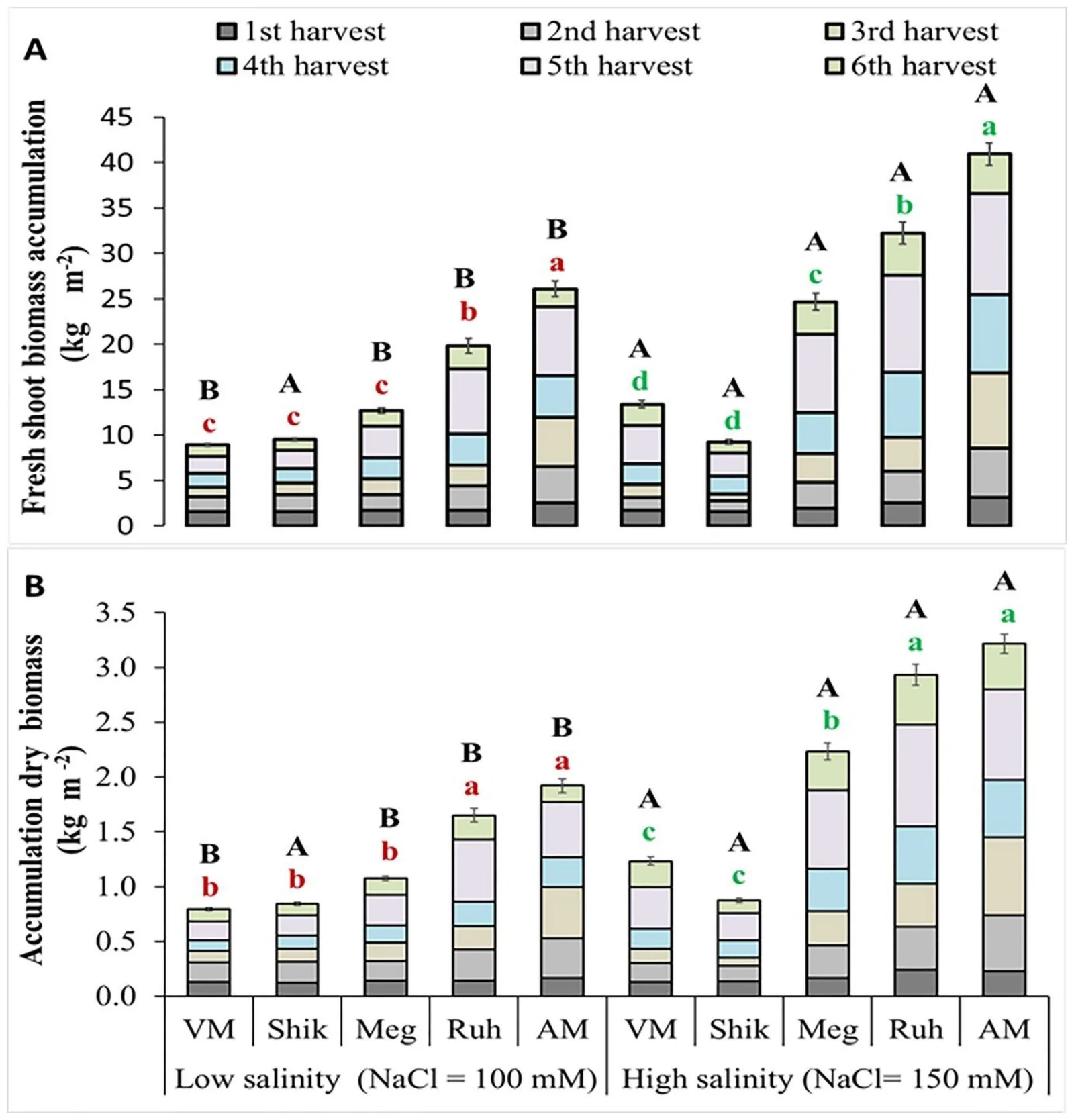
Fresh **(A)** and dry shoot weight **(B)** biomass accumulation of Sarcocornia (VM, Shikmona [Shik], Megadim [Meg], and Ruhama [Ruh]) and Arthrocaulon macrostachyum (AM). Plants were grown at one of two NaCl levels (100 and 150 mM). The means plus standard errors (± SE) are shown. Significant differences between salinity treatments of a particular ecotype are indicated by different uppercase letters, while significant differences between ecotypes under the same salinity treatment are represented by different lowercase letters (with identical colors), (Tukey’s HSD, p ≤ 0.05, n = 8).

Dry shoot biomass analysis indicated that increased salinity levels positively influenced dry biomass accumulation across all ecotypes, except Shik. Ruh and Meg showed the highest dry biomass accumulation at 150 mM NaCl. Overall, AM yielded greater dry biomass than the *Sarcocornia* ecotypes in all treatments, except for Ruh, which did not differ significantly from AM (**Figure 1B**).

Notably, the new ecotypes, particularly Ruh, showed the highest fresh and dry biomass at the lower salinity level (100 mM NaCl). Additionally, Ruh and Meg produced greater yields under higher salinity levels than the currently cultivated VM ecotype, whereas Shik produced yields comparable to VM at both salinity levels (**Figure 1**).

### 3.2 Effect of storage delay for one and two hours on water loss in harvested shoots

Halophytic plants, such as *Sarcocornia*, have high tissue water content due to their succulence. Therefore, delays between harvest and storage can lead to water loss, negatively affecting product quality (Gidado et al., 2024; Lufu et al., 2020). The current study examined the impacts of one- and two-hour storage delays on water loss.

Generally, plants of all ecotypes grown under lower salinity (100 mM) conditions experienced greater water loss than those grown under higher salinity (150 mM), except for the Shik ecotype, which showed a different pattern after a one-hour delay at lower salinity. The Ruh ecotype consistently showed the highest water loss across most treatments and delay durations, except for AM under lower salinity at both the one- and two-hour delay periods. In contrast, the Shik ecotype had lower water loss than the VM and AM after a one-hour delay in lower salinity conditions. Additionally, VM, Meg, and AM did not show significant differences in water loss between the one- and two-hour delay periods for either salinity treatment (**Figures 2A, B**).

**Figure 2:**
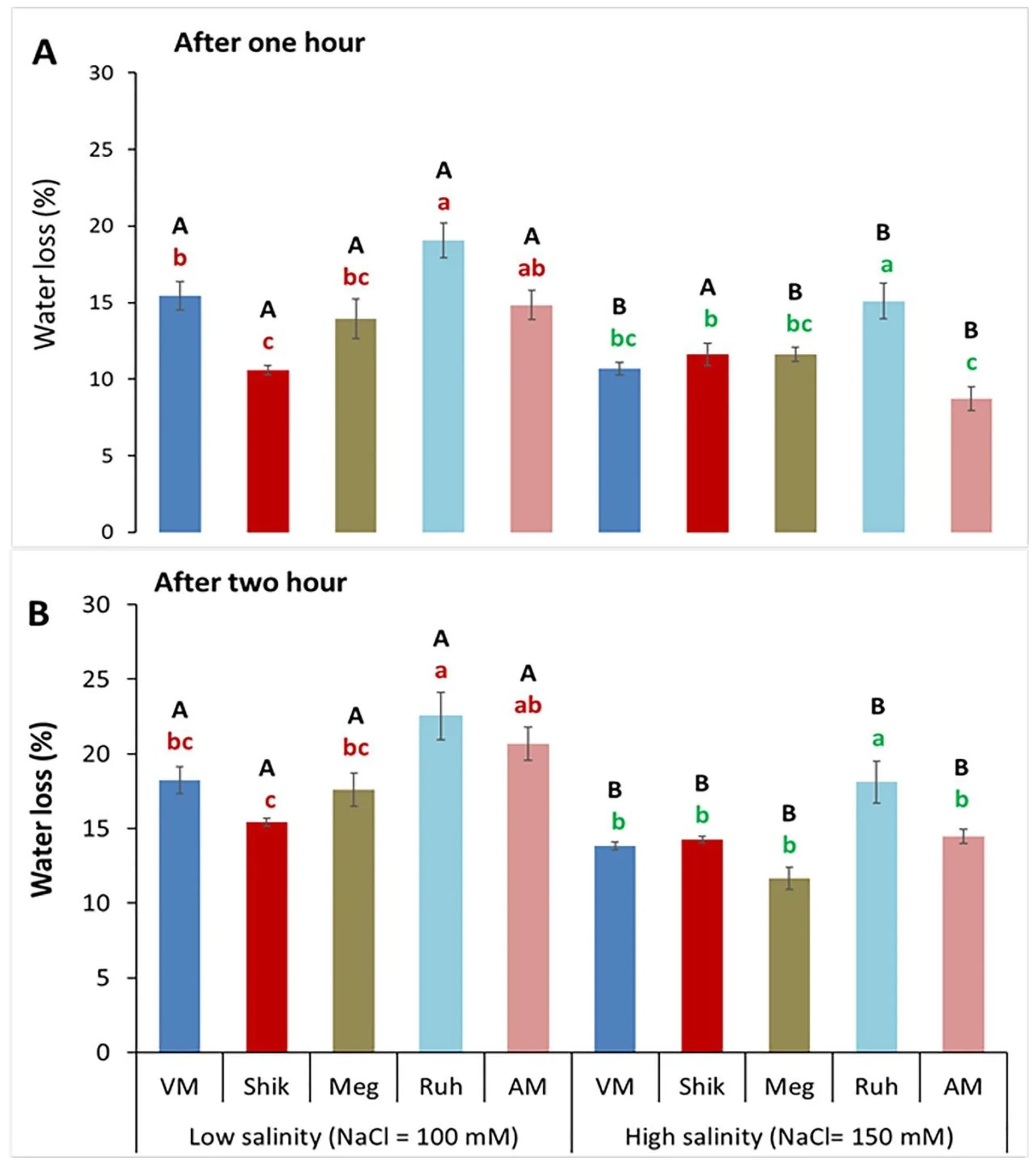
Water loss (%) after one **(A)** and two hours **(B)** of storage delay in Sarcocornia (VM, Shikmona [Shik], Megadim [Meg], and Ruhama [Ruh]) and Arthrocaulon macrostachyum (AM) harvested shoot samples. Plants were grown at one of two NaCl levels (100 and 150 mM). The means plus standard errors (± SE) are shown. Significant differences between salinity treatments of a particular ecotype are indicated by different uppercase letters, while significant differences between ecotypes under the same salinity treatment are represented by different lowercase letters (with identical colors), (Tukey’s HSD, p ≤ 0.05, n = 10). The presented results are representative of two independent experiments with similar results.

### 3.3 The effect of one- and two-hour storage delays on total carotenoid contents

The synthesis of small non-enzymatic molecules with antioxidant properties, such as flavonoids, anthocyanins, carotenoids, and polyphenolic compounds, constitutes a vital detoxification strategy for plants under stress conditions (Türkan and Demiral, 2009; Zhu, 2001), while ultimately being responsible for the quality of vegetable products (Maggio et al., 2011). Postharvest storage delays of one and two hours significantly increased the total carotenoid content in the *Sarcocornia* ecotypes grown under low salinity, but not in AM, for which carotenoid levels decreased after one hour but returned to control levels after two hours (**Figure 3**). Under high salinity, both AM and *Sarcocornia* ecotypes exhibited significant increases in carotenoid content following storage delays. Overall, the *Sarcocornia* ecotypes showed higher carotenoid levels than AM at both salinity levels and storage durations, relative to the immediately stored controls (**Figure 3**).

**Figure 3:**
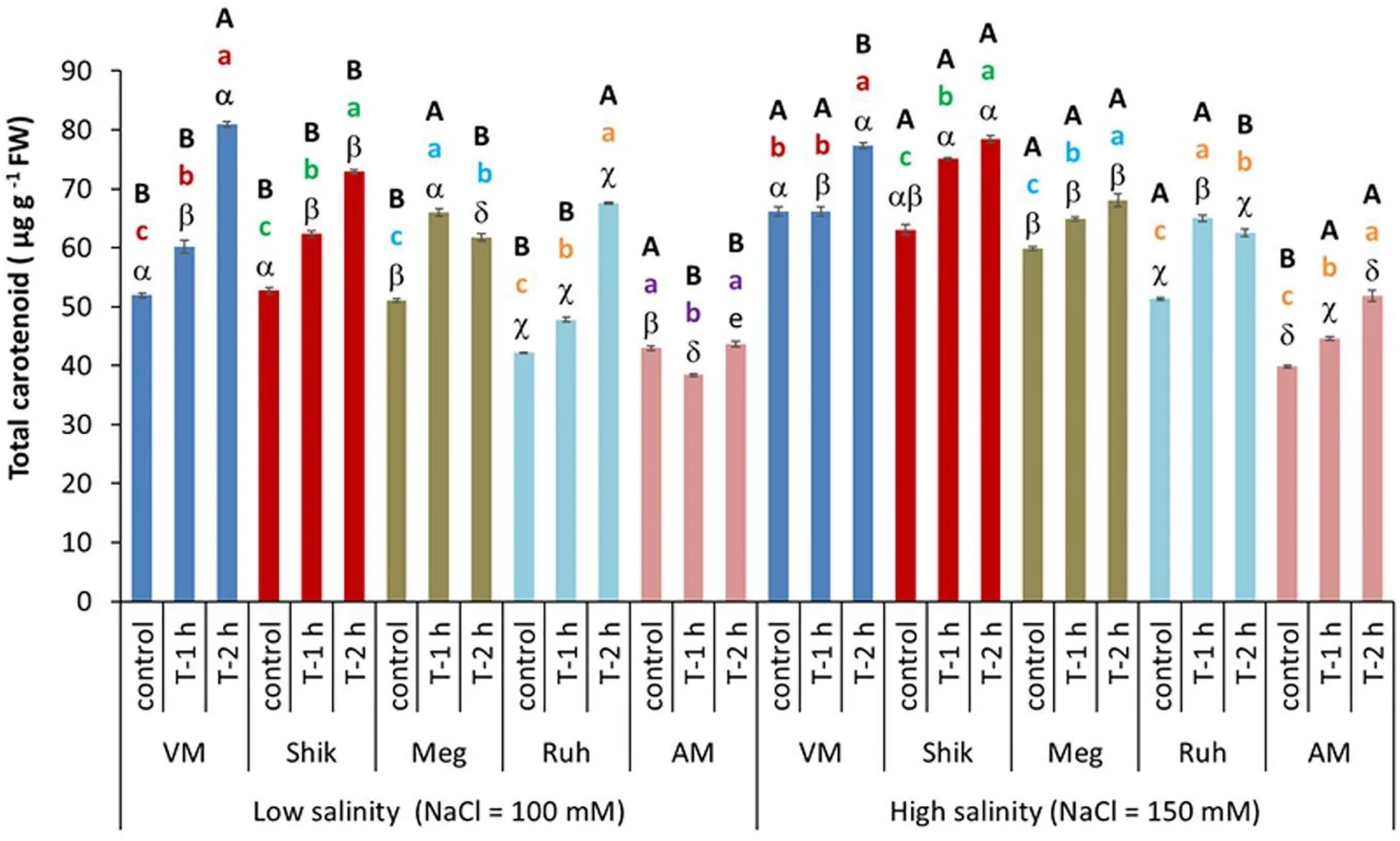
Total carotenoid content was measured immediately after harvest without storage delay (control), and after 1 h (T-1h) and 2 h (T-2h) of storage delay in *Sarcocornia* ecotypes (VM, Shikmona [Shik], Megadim [Meg], and Ruhama [Ruh]) and *Arthrocaulon macrostachyum* (AM). Plants were irrigated with two salinity levels (100 and 150 mM NaCl). Data are presented as means ± standard error (SE). Significant differences between salinity treatments within each ecotype with each specific storage treatment are indicated by different uppercase letters (black bars). Different lowercase letters indicate significant differences in storage duration (control, T-1h, and T-2h) within the same ecotype and salinity level (identical colors). Greek letters denote significant differences between ecotypes (VM, Shik, Meg, Ruh, and AM) under the same storage duration and salinity conditions (Tukey’s HSD, *p* ≤ 0.05, n = 6). The presented results are representative of two independent experiments with similar results.

### 3.4 The effect of one- and two-hour storage delays on total polyphenol content

Postharvest stress has been shown to enhance polyphenol biosynthesis in plants (Antunes et al., 2021). Delaying storage significantly increased the total polyphenol content in at least one of the two delay treatments across all *Sarcocornia* ecotypes, compared with the immediately stored controls. In contrast, the AM ecotype displayed reduced polyphenol content at both salinity levels. The interaction between salinity and storage delay varied among the ecotypes. VM, Shik, and Meg consistently maintained higher polyphenol levels under increased salinity and longer storage delays, with VM and Meg also showing elevated levels under low salinity conditions. In contrast, after two hours of storage delay, the Ruh ecotype exhibited lower polyphenol content under higher salinity than the control, but the content increased after one hour of storage delay under both salinity conditions and after two hours at lower salinity (**Figure 4**).

**Figure 4:**
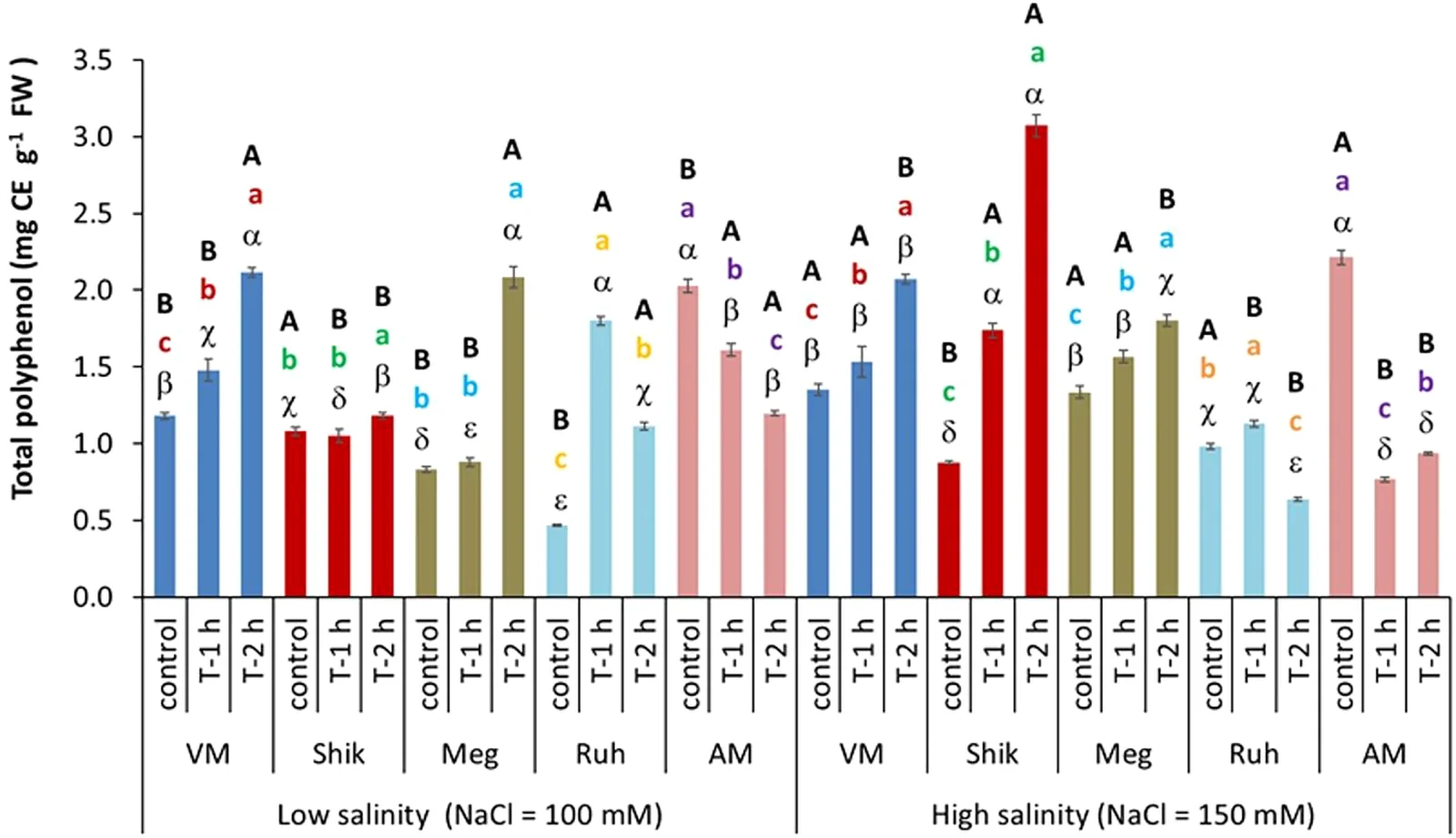
Shoot total polyphenol content was measured immediately after harvest without storage delay (control), and after 1 h (T-1h) and 2 h (T-2h) of storage delay in *Sarcocornia* ecotypes (VM, Shikmona [Shik], Megadim [Meg], and Ruhama [Ruh]) and *Arthrocaulon macrostachyum* (AM). Plants were irrigated with two salinity levels (100 and 150 mM NaCl). Data are presented as means ± standard error (SE). Significant differences between salinity treatments within each ecotype with each specific storage treatment are indicated by different uppercase letters (black bars). Different lowercase letters indicate significant differences between storage durations (control, T-1h, and T-2h) within the same ecotype and salinity level (identical color). Greek letters denote significant differences between ecotypes (VM, Shik, Meg, Ruh, and AM) under the same storage duration and salinity conditions (Tukey’s HSD, *p* ≤ 0.05, n = 6). The presented results are representative of two independent experiments with similar results.

Under control and one-hour storage delay conditions, VM, Meg, and Ruh exhibited higher polyphenol levels at high salinity than at low salinity. However, this trend reversed after a two-hour storage delay. Under high salinity and a two-hour storage delay, Shik exhibited the highest polyphenol content among all ecotypes (**Figure 4**).

### 3.5 The effect of one- and two-hour storage delays on total flavonoid contents

The total flavonoid content significantly increased in all *Sarcocornia* ecotypes after one or two hours of storage delay compared with immediately stored controls, except for Meg under lower salinity at two hours. In contrast, AM showed a reduction in flavonoid content under the storage delay conditions. The interaction between salinity and storage delay varied among ecotypes. VM and Meg consistently maintained elevated flavonoid levels under higher salinity and longer storage delays. In contrast, the flavonoid contents in VM, Shik, Meg, and Ruh were lower after a two-hour storage delay than after 1 hour, under low salinity, as were the levels in Shik and Ruh under high salinity (**Figure 5**).

**Figure 5:**
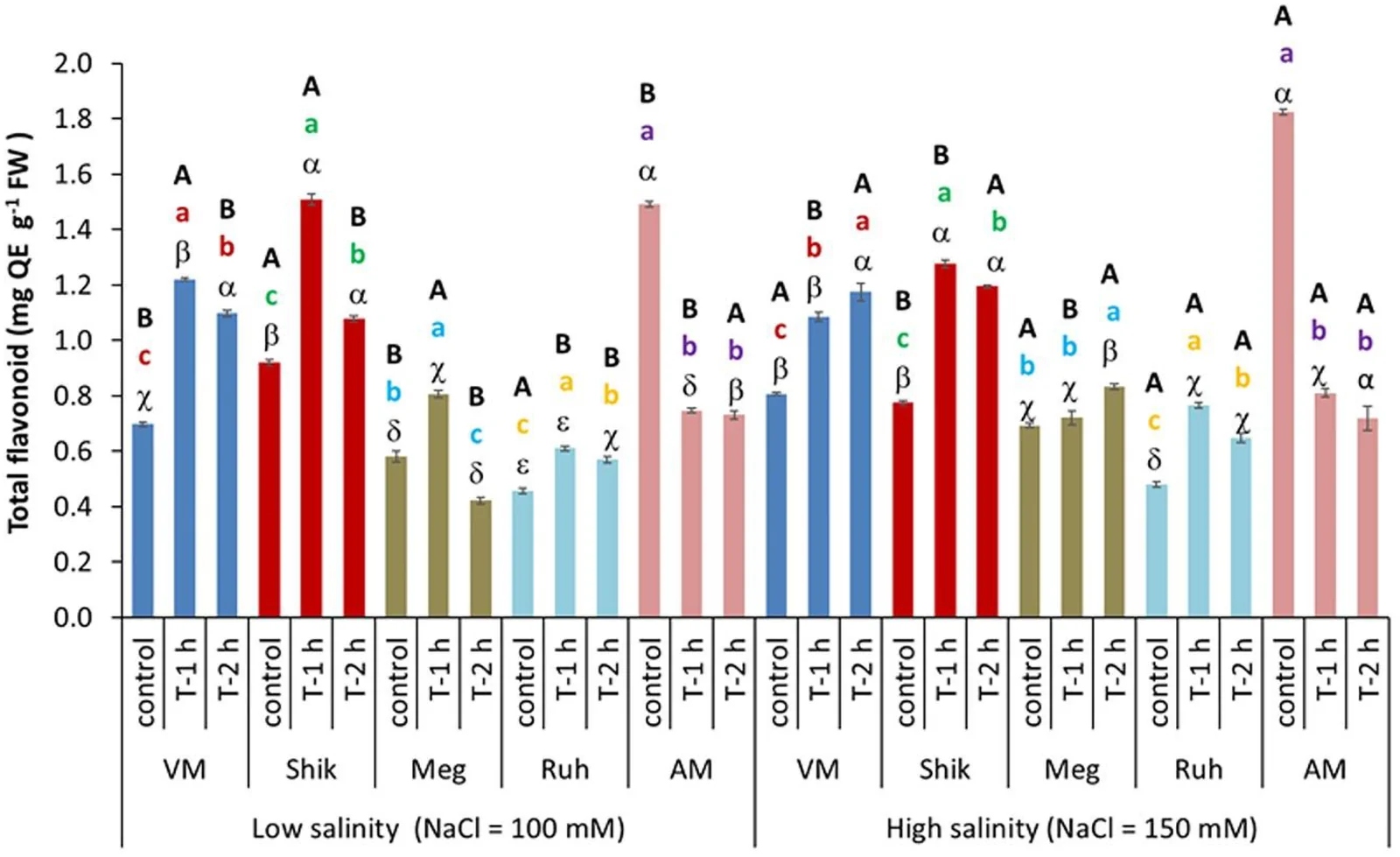
Total flavonoid content was measured immediately after harvest without storage delay (control), and after 1 h (T-1h) and 2 h (T-2h) of storage delay in *Sarcocornia* ecotypes (VM, Shikmona [Shik], Megadim [Meg], and Ruhama [Ruh]) and *Arthrocaulon macrostachyum* (AM). Plants were irrigated with two salinity levels (100 and 150 mM NaCl). Data are presented as means ± standard error (SE). Significant differences between salinity treatments within each ecotype with each specific storage treatment are indicated by different uppercase letters (black bars). Different lowercase letters indicate significant differences between storage durations (control, T-1h, and T-2h) within the same ecotype and salinity level (identical color). Greek letters denote significant differences between ecotypes (VM, Shik, Meg, Ruh, and AM) under the same storage duration and salinity conditions (Tukey’s HSD, *p* ≤ 0.05, n = 6). The presented results are representative of two independent experiments with similar results.

Under the control conditions and after a two-hour storage delay, VM, Meg, and Ruh had significantly higher total flavonoid content under high salinity than under low salinity. However, after a 1-hour storage delay, this trend reversed, with greater flavonoid accumulation observed under low-salinity conditions. Notably, under control conditions, AM exhibited the highest total flavonoid content among all the *Sarcocornia* ecotypes (**Figure 5**).

### 3.6 The effect of one- and two-hour storage delays on anthocyanin contents

In a previous study, anthocyanin levels were shown to increase in halophytic plants irrigated with highly saline water (Sisay et al., 2022). Similarly, in the current study, anthocyanin accumulation generally increased with higher salinity levels and extended storage durations. One and two-hour storage delays significantly elevated the anthocyanin levels in both ecotypes and across salinity treatments, compared with the immediately stored controls. In most ecotypes, anthocyanin content increased progressively over the first two hours of storage, except for Meg and AM under low salinity and Shik under high salinity (**Figure 6**).

**Figure 6:**
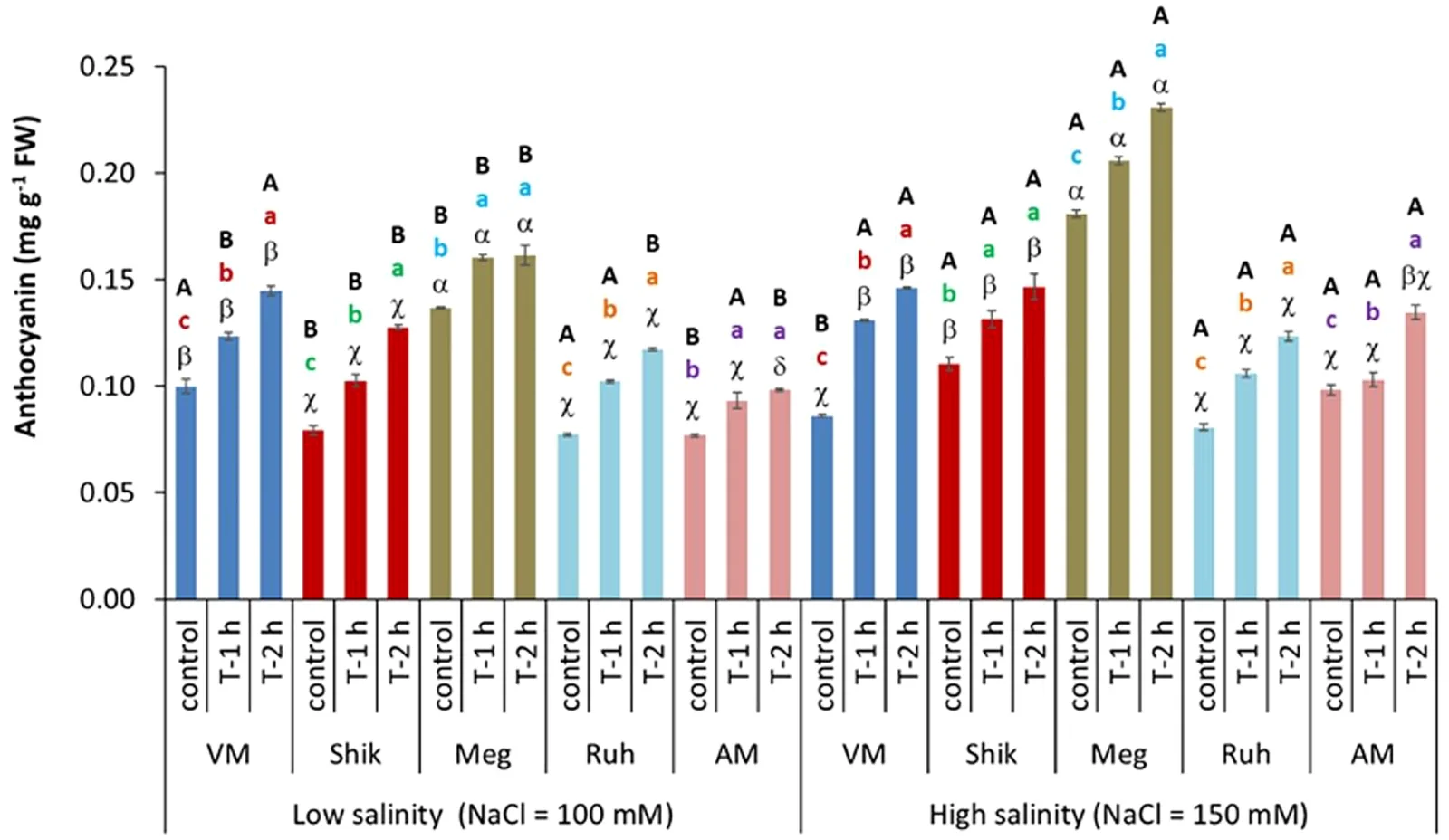
Total anthocyanin content was measured immediately after harvest without storage delay (control), and after 1 h (T-1h) and 2 h (T-2h) of storage delay in *Sarcocornia* ecotypes (VM, Shikmona [Shik], Megadim [Meg], and Ruhama [Ruh]) and *Arthrocaulon macrostachyum* (AM). Plants were irrigated with two salinity levels (100 and 150 mM NaCl). Data are presented as means ± standard error (SE). Significant differences between salinity treatments within each ecotype with each specific storage treatment are indicated by different uppercase letters (black bars). Different lowercase letters indicate significant differences in storage duration (control, T-1h, and T-2h) within the same ecotype and salinity level (identical colors). Greek letters denote significant differences between ecotypes (VM, Shik, Meg, Ruh, and AM) under the same storage duration and salinity conditions (Tukey’s HSD, *p* ≤ 0.05, n = 3). The presented results are representative of two independent experiments with similar results.

Under high salinity conditions, the Shik and Meg ecotypes exhibited significantly higher anthocyanin levels than under low salinity conditions, including under the control and the one- and two-hour storage delay treatments. Additionally, the VM ecotype showed an increase in anthocyanin accumulation after a one-hour storage delay, whereas the Ruh and AM ecotypes showed an increase after a two-hour delay. Overall, the Meg ecotype had the highest anthocyanin accumulation across all ecotypes in both salinity treatments, whereas the Ruh and AM ecotypes displayed comparatively lower levels in these treatments (**Figure 6**).

### 3.7 The effect of one- and two-hour storage delays on total soluble sugar and electrical conductivity

Total soluble sugar and electrical conductivity (EC) are key physiological indicators that are commonly enhanced in halophytic plants under saline conditions, particularly with increasing NaCl concentrations (Ventura et al., 2011a; Ventura and Sagi, 2013). In halophytes such as *Salicornia europaea* and *Sarcocornia fruticosa*, elevated salinity has been associated with substantial accumulation of soluble sugars, with total soluble sugar levels reaching values of approximately 6.3% and 7.6%, respectively, contributing to improved osmotic adjustment and palatability (Ventura et al., 2011a; Ventura and Sagi, 2013, and references therein). In the current study, the total soluble sugar content significantly increased after one and two hours of storage delay across all ecotypes, compared with the control samples stored immediately after harvest. Extended storage delays led to even greater increases in total soluble sugar, particularly between one and two hours, at both salinity levels. However, this trend did not apply to the Ruh and AM ecotypes under high salinity conditions (**Figure 7A**).

**Figure 7:**
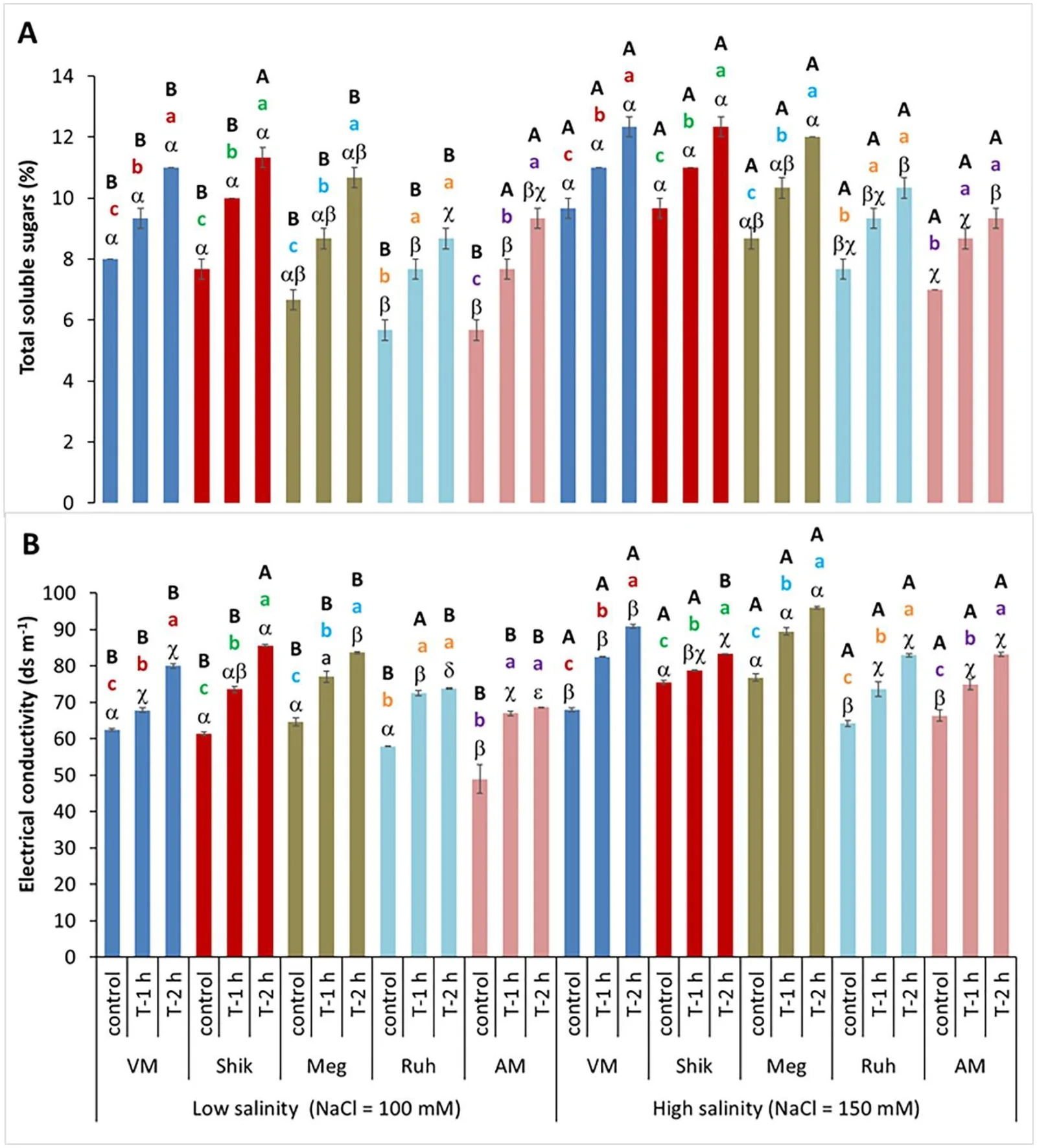
Total soluble sugar **(A)** and electrical conductivity **(B)** contents in plant shoots were measured immediately after harvest without storage delay (control), and after 1 h (T-1h) and 2 h (T-2h) of storage delay in *Sarcocornia* ecotypes (VM, Shikmona [Shik], Megadim [Meg], and Ruhama [Ruh]) and *Arthrocaulon macrostachyum* (AM). Plants were irrigated with two salinity levels (100 and 150 mM NaCl). Data are presented as means ± standard error (SE). Significant differences between salinity treatments within each ecotype with each specific storage treatment are indicated by different uppercase letters (black bars). Different lowercase letters indicate significant differences between storage durations (control, T-1h, and T-2h) within the same ecotype and salinity level (identical colors). Greek letters denote significant differences between ecotypes (VM, Shik, Meg, Ruh, and AM) under the same storage duration and salinity conditions (Tukey’s HSD, *p* ≤ 0.05, n = 3). The presented results are representative of two independent experiments with similar results.

Similar to total soluble sugar, the EC increased with longer storage delays. Generally, EC values were higher in the one- and two-hour storage delay samples than in the immediately stored control samples, although some ecotype-specific exceptions were observed. Accordingly, EC levels rose after one to two hours of storage delay; however, Ruh and AM did not show a significant difference between the one- and two-hour delays under low salinity conditions (**Figure 7B**). As for the effect of salinity, the total soluble sugar content increased significantly with rising salinity, except in the AM ecotype under both one- and two-hour storage delays and in the Shik ecotype after a two-hour storage delay (**Figure 7A**). Similarly, the EC increased significantly with increasing salinity, except for the Shik ecotype after a two-hour storage delay and the Ruh ecotype after a one-hour storage delay (**Figure 7B**).

### 3.8 The effect of one- and two-hour storage delays on radical scavenging activities

Radical scavenging activity represents a defense mechanism under various stress conditions, playing a vital role in neutralizing reactive oxygen species that contribute to lipid peroxidation and the effects of abiotic stress (Kumar et al., 2017; Patel et al., 2025).

Radical scavenging activity increased significantly following one- and two-hour storage delays in the *Sarcocornia* ecotypes at both salinity levels. In contrast, AM showed a significant reduction in radical scavenging activity over time, compared with the immediately stored control (**Figure 8**).

**Figure 8:**
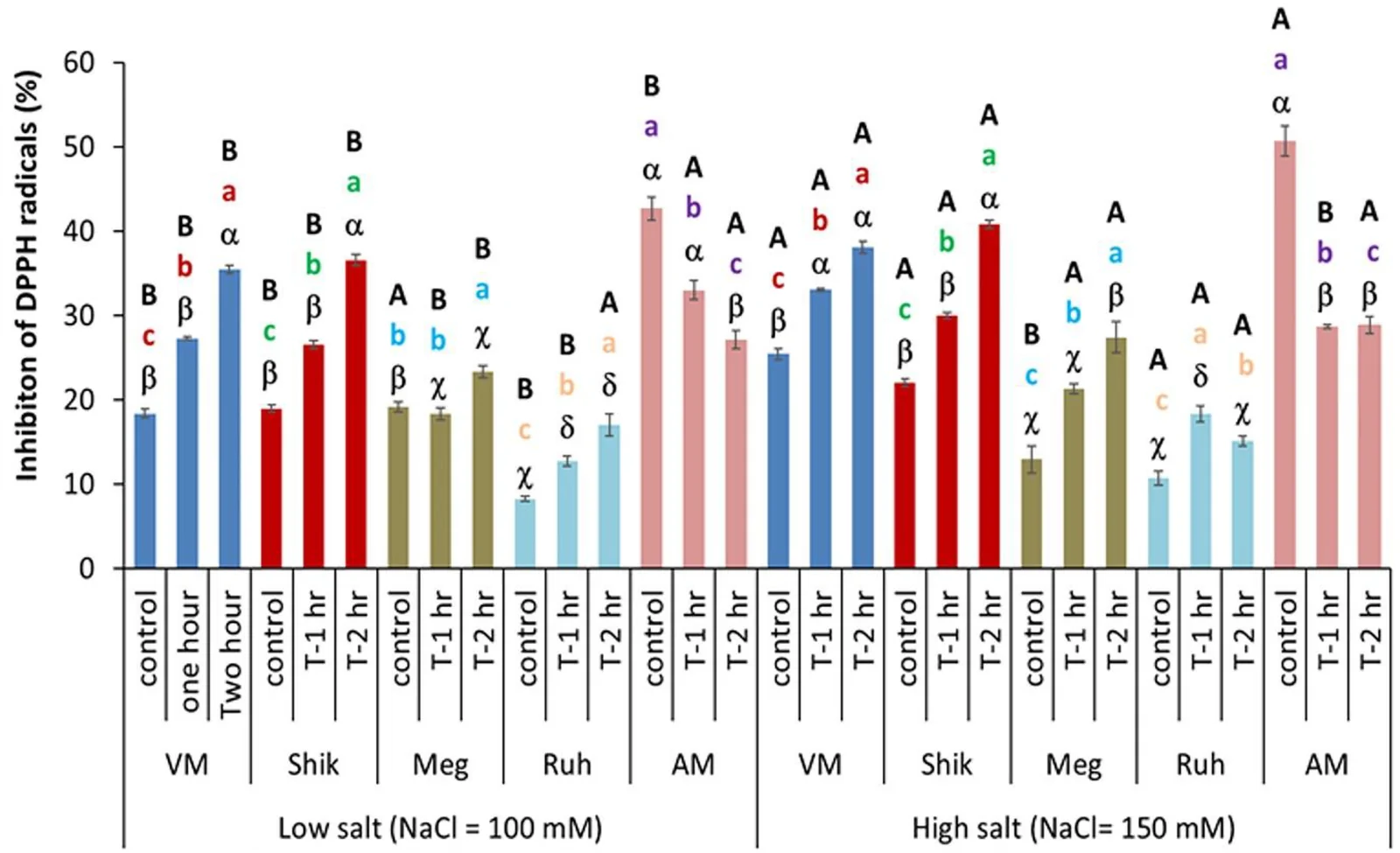
Radical scavenging activity by DPPH (2,2-diphenyl-1-picrylhydrazyl) was measured immediately after harvest without storage delay (control), and after 1 h (T-1h) and 2 h (T-2h) of storage delay in *Sarcocornia* ecotypes (VM, Shikmona [Shik], Megadim [Meg], and Ruhama [Ruh]) and *Arthrocaulon macrostachyum* (AM). Plants were irrigated with two salinity levels (100 and 150 mM NaCl). Data are presented as means ± standard error (SE). Significant differences between salinity treatments within each ecotype with each specific storage treatment are indicated by different uppercase letters (black bars). Different lowercase letters indicate significant differences in storage duration (control, T-1h, and T-2h) within the same ecotype and salinity level (identical colors). Greek letters denote significant differences between ecotypes (VM, Shik, Meg, Ruh, and AM) under the same storage duration and salinity conditions (Tukey’s HSD, *p* ≤ 0.05, n = 5). The presented results are representative of two independent experiments with similar results.

Higher salinity increased radical-scavenging activity in the *Sarcocornia* ecotypes after one and two-hour storage delays. Among the ecotypes, Ruh consistently exhibited lower radical scavenging activity than the others at both salinity levels. Notably, although the AM ecotype showed a decrease in radical scavenging activity, its levels remained higher than those of the Ruh and Meg ecotypes at lower salinity and exceeded those of the Ruh ecotype at higher salinity (**Figure 8**).

### 3.9 The effect of one- and two-hour storage delays on malondialdehyde content

Malondialdehyde (MDA) is a product of lipid peroxidation during oxidative stress in plant tissue, and thus, it is characteristic of naturally senescent plant parts (Yarmolinsky et al., 2013, and references therein). Above a certain level, MDA can be toxic to plant tissue and needs further detoxification (Patel et al., 2025; Srivastava et al., 2017).

MDA levels increased with storage delay duration in most ecotypes and salinity treatments, compared to the immediately stored control samples. An exception was observed in the AM ecotype under low salinity: MDA levels did not increase after a one-hour storage delay, but they did after a two-hour delay (**Figure 9**).

**Figure 9:**
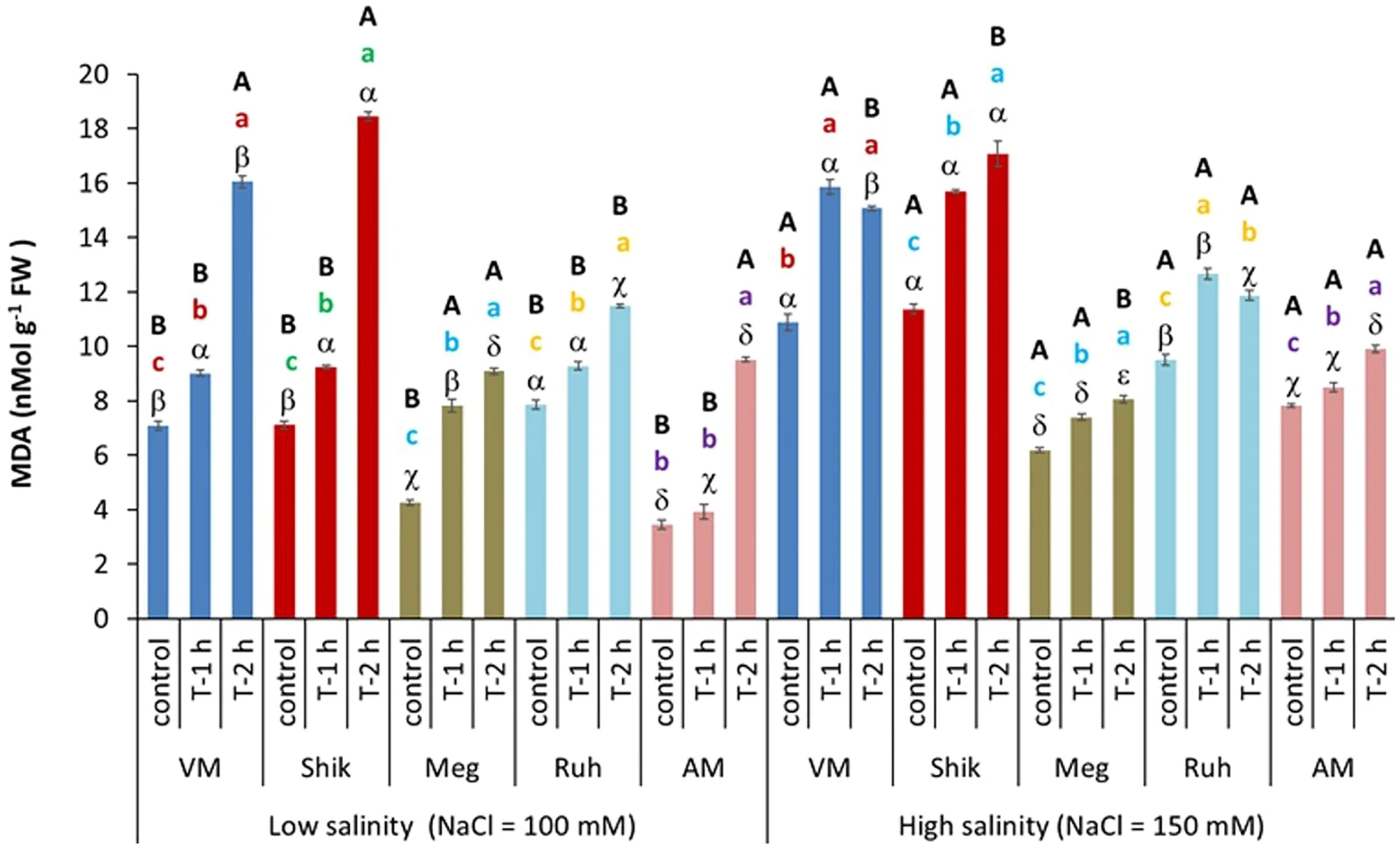
Malondialdehyde (MDA) content was measured immediately after harvest without storage delay (control), and after 1 h (T-1h) and 2 h (T-2h) of storage delay in *Sarcocornia* ecotypes (VM, Shikmona [Shik], Megadim [Meg], and Ruhama [Ruh]) and *Arthrocaulon macrostachyum* (AM). Plants were irrigated with two salinity levels (100 and 150 mM NaCl). Data are presented as means ± standard error (SE). Significant differences between salinity treatments within each ecotype with each specific storage treatment are indicated by different uppercase letters (black bars). Different lowercase letters indicate significant differences in storage duration (control, T-1h, and T-2h) within the same ecotype and salinity level (identical colors). Greek letters denote significant differences between ecotypes (VM, Shik, Meg, Ruh, and AM) under the same storage duration and salinity conditions (Tukey’s HSD, *p* ≤ 0.05, n = 4). The presented results are representative of two independent experiments with similar results.

Under control and one-hour storage delay conditions, the VM, Shik, Ruh, and AM ecotypes exhibited significantly higher MDA levels under high salinity than under low salinity. However, after a 2-hour storage delay, VM and Shik showed elevated MDA levels in low-salinity conditions compared to high-salinity conditions (**Figure 9**).

MDA levels in VM, Shik, Meg, and AM increased progressively from one to two hours of storage delay for both salinity treatments. In contrast, VM and Ruh exhibited a decrease in MDA levels after two hours of storage delay under high salinity. Notably, the highest MDA levels were recorded in VM and Shik under high salinity, with both 1- and 2-hour storage delays, and under low salinity with the two-hour delay (**Figure 9**).

### 3.10 The impact of a two-hour storage delay and subsequent 21-day storage on water loss in harvested plant shoots

In addition to the water loss that occurs during the storage delay period, harvested vegetables continue to lose water during cold storage. Accordingly, water loss in shoots harvested from plants irrigated with 150 mM NaCl and immediately placed into 21-day cold storage (control) was compared with that in shoots subjected to a two-hour storage delay and with shoots subjected to a two-hour delay followed by 21 days of cold storage.

Water loss increased progressively across treatments, from the 21-day stored control to shoots exposed to a two-hour delay, to shoots stored for 21 days after a two-hour delay. The highest water loss was observed in shoots subjected to a two-hour delay followed by 21 days of storage. Except for the VM ecotype, which exhibited relatively low water loss during both the two-hour delay and the combined two-hour delay plus 21-day storage treatments, Ruh showed the lowest water loss among the ecotypes in the 21-day control treatment. Notably, the smallest increase in water loss between immediate storage and storage following a two-hour delay was observed in VM, whereas the other *Sarcocornia* ecotypes and AM exhibited greater water loss (**Figure 10**).

**Figure 10:**
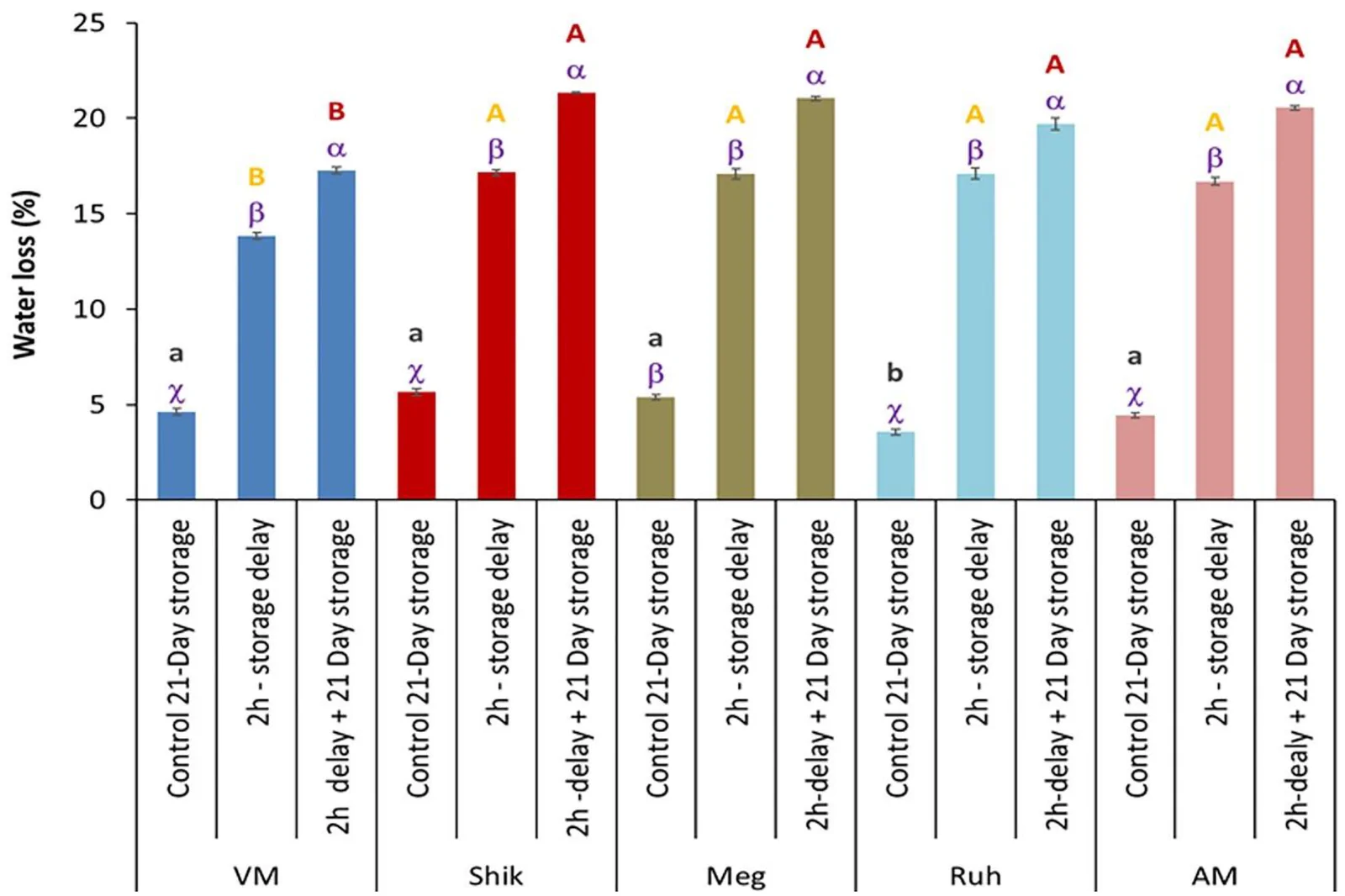
Comparison of water loss (%) in freshly harvested shoots exposed to a two-hour storage delay with and without 21-day cold room storage and the control of 21-day storage without storage delay of the Sarcocornia ecotypes VM, Shikmona (Shik), Megadim (Meg), and Ruhama (Ruh), and of Arthrocaulon macrostachyum (AM). Plants irrigated with 150 mM NaCl were examined. The means plus standard errors (± SE) are shown. Significant differences between ecotypes treated with 2-h storage delay or 2-h storage delay plus 21-day cold storage treatments are indicated by different uppercase yellow and red letters, respectively. Different lowercase letters indicate significant differences between the control ecotypes after 21 days of cold storage (black letters). The different Greek letters indicate differences between the same ecotypes during a 2-h storage delay, with and without 21 days of storage treatment. (Tukey’s HSD, p ≤ 0.05, n = 3). The presented results are representative of two independent experiments with similar results.

### 3.11 The effect of 21-day storage, with and without a two-hour storage delay, on the levels of total carotenoids, total flavonoids, total polyphenols, and DPPH radical inhibition activity

The synthesis of non-enzymatic antioxidant and bioactive compounds, including carotenoids, flavonoids, and polyphenols, is essential for antioxidant detoxification under oxidative stress and contributes to the nutritional quality of plant-based products (Harboub et al., 2025; Maggio et al., 2011; Türkan and Demiral, 2009; Zhu, 2001). To evaluate whether storage delays affect antioxidant capacity, these compounds were quantified in harvested *Sarcocornia* ecotypes and AM after 21 days of storage, with and without a two-hour delay.

Total carotenoid content was significantly higher in shoots stored for 21 days after a two-hour delay, compared with shoots stored immediately after harvest, followed by 21 days in a cold room, both in the *Sarcocornia* ecotypes and in AM (**Figure 11A**). In VM, Shik, and Meg, the increase in carotenoid content per fresh weight (≥ 40%) exceeded the reduction attributed to water loss (12.64–15.96%; **Figures 10**, **11A**). In contrast, Ruh and AM showed smaller increases in carotenoid content [4.12% and 11.87%, respectively (**Figure 11A**)].

**Figure 11:**
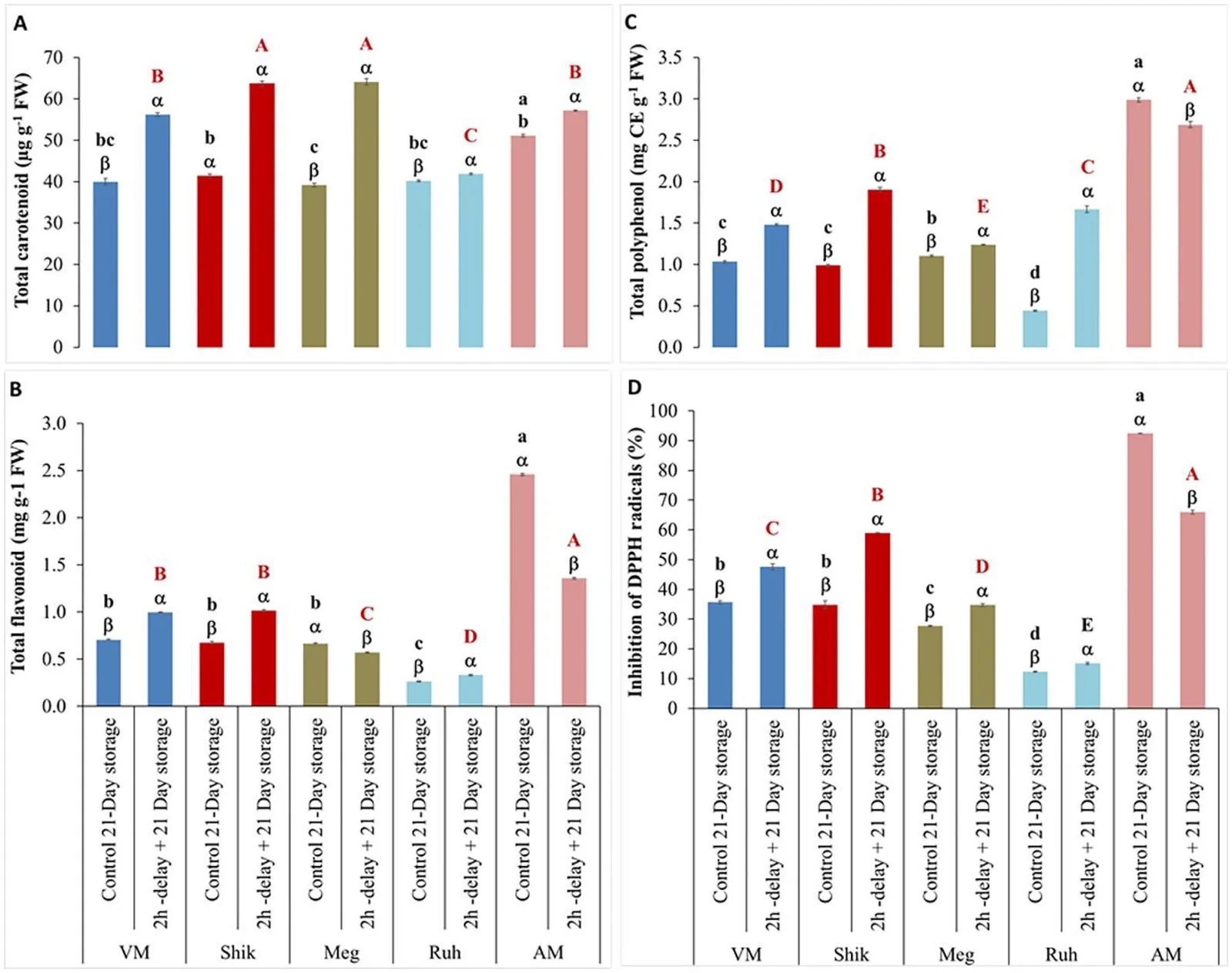
The effect of 21-day cold storage after a postharvest two-hour (2 h) storage delay, as well as immediate storage as the control, on total carotenoid **(A)**, total flavonoid **(B)**, total polyphenol **(C)**, and radical scavenging activities as measured by DPPH (2,2-diphenyl-1-picrylhydrazyl) level **(D)** in freshly harvested shoots of Sarcocornia fruticosa VM, Shikmona (Shik), Megadim (Meg), and Ruhama (Ruh) ecotypes, as well as Arthrocaulon macrostachyum (AM), irrigated with water containing 150 mM NaCl. The means plus standard errors (± SE) are shown. Significant differences between ecotypes treated with a 2-h storage delay and 21-day cold storage treatments are indicated by different uppercase red letters. Different lowercase black letters indicate significant differences between ecotypes exposed to the control (21-day cold storage without delay). The different Greek letters indicate significant differences between the two treatments in the same ecotype (control vs. treated samples). (Tukey’s HSD, p ≤ 0.05, n = 6). The presented results are representative of two independent experiments with similar results.

Total flavonoid content increased significantly in VM, Shik, and Ruh, but decreased in Meg and AM. The increases observed in VM and Shik (40.8% and 52.2%, respectively; **Figures 10**, **11B**) exceeded the water loss. Ruh showed a flavonoid increase comparable to its water loss, whereas Meg and AM showed decreases, with AM exhibiting a pronounced 44.7% reduction after a two-hour delay followed by 21 days of storage in a cold room (**Figures 10**, **11B**).

Total polyphenol content increased modestly in Meg (12.72%), closely corresponding to its water loss. In contrast, VM, Shik, and Ruh exhibited substantially greater increases in polyphenol content (43.68%, 91.99%, and 279.54%, respectively), which exceeded the decrease in water loss and indicated a positive effect of the two-hour delay before a 21-day storage in a cold room (**Figures 10**, **11C**).

All *Sarcocornia* ecotypes exhibited significantly greater inhibition of DPPH radicals following a two-hour storage delay before a 21-day cold room storage, compared with immediate storage (VM: 33.40%, Shik: 69.20%, Meg: 25.30%, Ruh: 22.54%), exceeding the corresponding reductions in water loss (**Figures 10**, **11D**). In contrast, AM exhibited significant decreases in total flavonoids, total polyphenols, and radical scavenging activity following the two-hour delay and subsequent 21-day storage, indicating that immediate storage is preferable for AM (**Figures 11B–D**).

### 3.12 Effect of a two-hour storage delay on malondialdehyde content after 21-day storage

Malondialdehyde (MDA), a byproduct of lipid peroxidation, is linked to prolonged oxidative stress and natural aging in plant tissues (Yarmolinsky et al., 2013). In this study, delaying storage for 2 hours before subjecting harvested shoots to 21 days of cold storage significantly increased MDA content compared to those shoots that were immediately stored for the full 21 days across all examined ecotypes (**Figure 12**).

**Figure 12:**
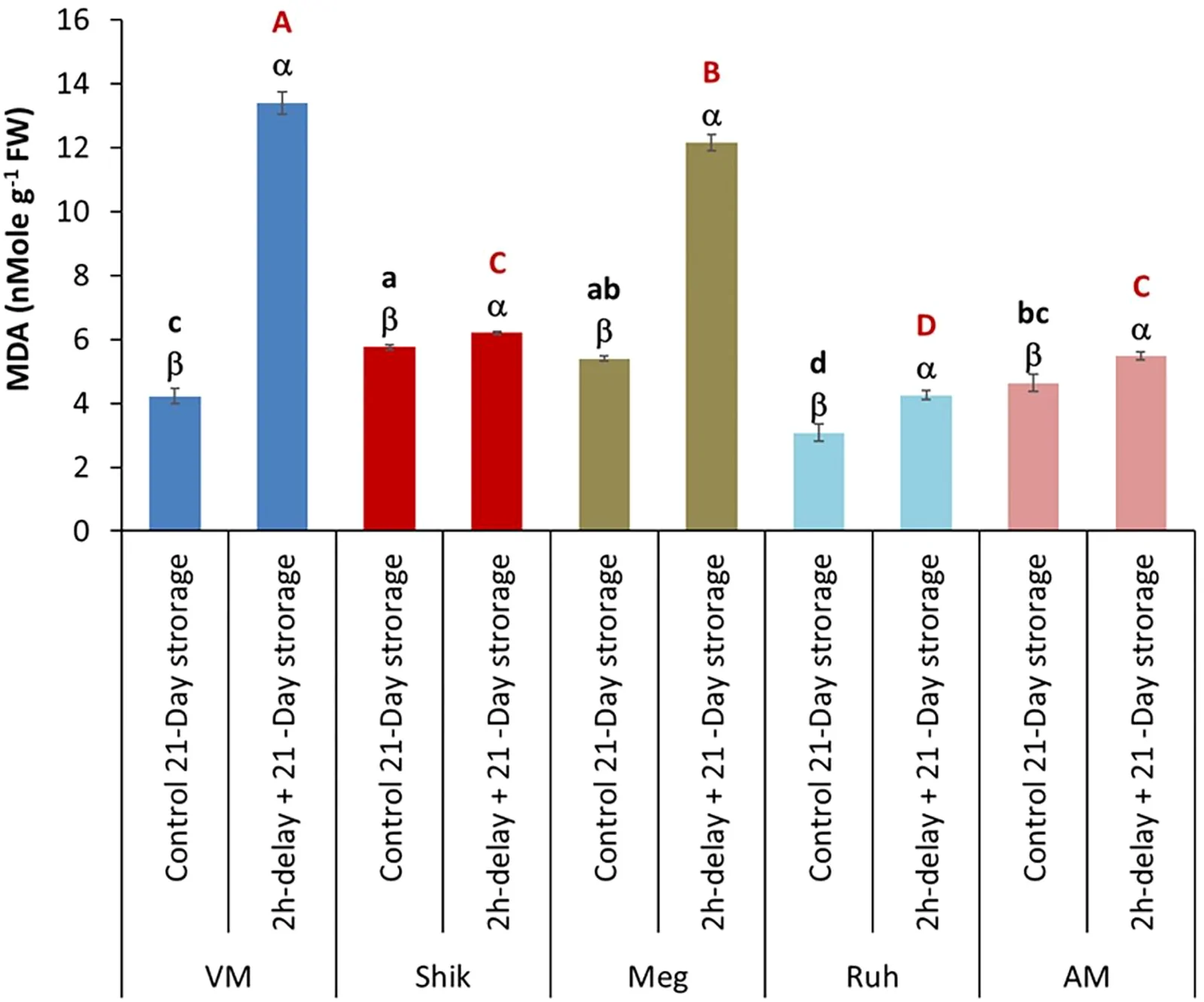
The effect of 21-day cold storage after a postharvest two-hour (2 h) storage delay, as well as direct storage without any delay as the control, on malondialdehyde contents in freshly harvested shoots of Sarcocornia fruticosa VM, Shikmona (Shik), Megadim (Meg), and Ruhama (Ruh) ecotypes, as well as Arthrocaulon macrostachyum (AM) irrigated with water containing 150 mM NaCl. The means plus standard errors (± SE) are shown. Significant differences between ecotypes treated with a 2-h storage delay and 21-day cold storage treatments are indicated by different uppercase red letters. Different lowercase black letters indicate significant differences between ecotypes exposed to the control (21- day cold storage without delay). The different Greek letters indicate a significant difference between the two treatments in the same ecotype (control vs. treated samples). (Tukey’s HSD, p ≤ 0.05, n = 3). The presented results are representative of two independent experiments with similar results.

Under the two-hour storage delay plus 21-day storage treatment, VM and Meg exhibited the highest MDA levels, whereas Ruh showed the lowest. In contrast, under the control treatment (immediate storage), Shik exhibited the highest MDA concentration among the ecotypes, except for Meg, whereas Ruh showed the lowest MDA content (**Figure 12**).

## 4 Discussion

In conventional leafy vegetables, reductions in antioxidant capacity, phenolic compounds, and visual quality are commonly reported with increasing storage duration and water loss [dehydration (Galani et al., 2017; Gil et al., 1999)]. Similar degradative trends have been observed in the halophytic species *Sarcocornia perennis* and *Salicornia ramosissima*, including discoloration and quality loss during extended cold storage (Gago et al., 2011).

The current study addresses a critical knowledge gap by examining the effects of short postharvest storage delays (one and two hours) on the nutritional quality of *Sarcocornia fruticosa* ecotypes and one AM ecotype grown under lower and higher salinity (100 and 150 mM NaCl). Unlike the deterioration typically observed in conventional vegetables, these short storage delays consistently enhanced several nutritional parameters in the studied halophytes. Notably, many of these improvements exceeded what could be attributed solely to water loss, suggesting that active physiological and metabolic processes occur in response to postharvest dehydration.

Lower salinity (100 mM NaCl) resulted in greater water loss than higher salinity (150 mM NaCl) in most ecotypes, suggesting better water retention at higher salinity (**Figure 2A**). This response may be due to osmotic adjustment and tissue succulence, which help halophytes maintain cellular hydration in saline environments. However, the Shik ecotype showed no significant difference in water loss after one hour between the two salinity levels (**Figure 2A**).

The Ruh ecotype experienced the highest water loss at both one and two hours across the two salinity levels, suggesting that it has a lower capacity for short-term water conservation during postharvest storage delays. In contrast, the AM ecotype at lower salinity showed no significant difference in water loss compared to the Ruh ecotype at both one and two hours of storage delay. Overall, the reduced water loss observed at higher salinity levels indicates physiological adaptations that enhance water-holding capacity and postharvest stability (**Figures 2A, B**).

A schematic summarizing response under control, one-hour, and two-hour postharvest delay treatments, integrating antioxidant compounds, osmoprotective metabolites, and oxidative stress indicators, was generated to facilitate comparison among ecotypes and to improve the interpretation of postharvest responses (**Supplementary Figure 5**). The schematic illustrates that *Sarcocornia fruticosa* ecotypes generally enhanced their levels of total polyphenols, flavonoids, and radical scavenging activity following one- and two-hour postharvest storage delays, indicating activation of antioxidant defense mechanisms in response to dehydration stress. In contrast, *Arthrocaulon macrostachyum* showed reductions in these antioxidant traits after both 1- and 2-hour storage delays. In both species, carotenoids, anthocyanins, total soluble solids, and EC increased following postharvest delay, suggesting enhanced accumulation of protective metabolites and changes in cellular osmotic status during early water-loss.

### 4.1 Postharvest storage delays enhanced total soluble sugar and electrical conductivity levels in *Sarcocornia fruticosa* ecotypes and *Arthrocaulon macrostachyum*

Postharvest dehydration imposes osmotic stress, triggering osmotic adjustment processes in plant tissues (Lufu et al., 2020). In halophytes, drought and salinity stress commonly induce the accumulation of soluble sugars and ion redistribution, reflected in increased TSS and EC (Al Hassan et al., 2016). These changes contribute to osmotic balance, improved palatability, and extended shelf life (Antunes et al., 2021; Ksouri et al., 2012; Ventura et al., 2011a).

Total soluble sugar content significantly increased after one- and two-hour delays across most ecotypes and salinity treatments compared with the control samples (**Figure 7A**; **Supplementary Figures 1A, B**). After a one-hour storage delay, total soluble sugar levels in most *Sarcocornia* ecotypes and AM remained close to the values expected from water loss alone, indicating limited early metabolic adjustment (**Figure 7A**; **Supplementary Figure 1A**). However, the Shik ecotype by 16.68% under lower salinity and AM by 13.03% under higher salinity showed greater increases in total soluble sugar than expected given water loss, suggesting faster activation of osmotic regulation mechanisms and more efficient mobilization or synthesis of soluble sugars during short-term dehydration.

After a two-hour storage delay, total soluble sugar levels exceeded the values expected based on water-loss reduction in most *Sarcocornia* ecotypes under the lower salinity treatment, including VM (12.47%), Shik (25.05%), Meg (31.89%), and Ruh (18.44%), as well as in AM (30.6%). Under the higher salinity treatment, total soluble sugar levels were also markedly higher than expected in Meg (22.32%) and AM (13.91%) relative to the expected after water-loss reduction (**Figure 7A**; **Supplementary Figure 1B**). This pattern suggests that prolonged water loss consistently stimulates carbohydrate accumulation and enhances the metabolic pathways associated with osmoprotection, membrane stabilization, and dehydration tolerance (Kumar et al., 2017; Sisay et al., 2022; Ventura et al., 2014). In contrast, under higher salinity, VM, Shik, and Ruh maintained total soluble sugar concentrations similar to the expected post-water-loss baseline, reflecting more stable soluble sugar dynamics under salt-enriched conditions (**Supplementary Figure 1B**).

Electrical conductivity increased significantly after one- and two-hour delays across most ecotypes and salinity treatments, contrary to the expected results after water loss (**Figure 7B**; **Supplementary Figures 2A, B**). Under the lower salinity treatment, a one-hour delay resulted in an increase in EC for Shik, a decrease for VM, and no change for Meg, Ruh, and AM, which remained consistent with their expected water-loss responses. After two hours, EC increased in VM, Shik, and Meg by 6.2%, 18.06%, and 9.1%, respectively, while Ruh and AM continued to align with their anticipated water-loss responses (**Figure 7B**; **Supplementary Figures 2A, B**).

In the higher salinity treatment, EC levels exceeded the expected level considering water loss in VM and Meg by 8.4% and 5.6%, respectively, while Shik levels decreased by 8.1% after a one-hour delay. Ruh and AM remained consistent with their expected water-loss responses. After two hours, there was a significant increase in EC for VM (15.2%), Meg (10.34%), Ruh (5.7%), and AM (7.2%), whereas Shik exhibited a significantly decreased EC by 5.4% compared with its anticipated water-loss response (**Figure 7B**; **Supplementary Figures 2A, B**).

These increases largely exceeded expectations based on water loss alone and are attributed to moisture loss, enhanced soluble ion concentration, and rapid osmotic adjustment, which increase tissue ionic strength and maintain turgor under dehydration stress, thereby enhancing electrical conductivity (Sisay et al., 2022; Ventura et al., 2011a, 2014).

### 4.2 One- and two-hour storage delays enhanced malondialdehyde levels in harvested *Sarcocornia fruticosa* ecotypes and *Arthrocaulon macrostachyum*

Abiotic stresses, such as dehydration and salinity, induce oxidative stress by increasing the production of reactive oxygen species (ROS). This rise in ROS levels leads to lipid peroxidation (Kumar et al., 2021; Sagi et al., 2004; Soltabayeva et al., 2022). MDA, a toxic aldehyde produced during lipid peroxidation, is frequently used as an indicator of oxidative stress in plant tissues (Ali et al., 2017; Sahin et al., 2018; Patel et al., 2025).

In this study, MDA levels increased after one- and two-hour storage delays compared with control plant samples across all ecotypes and salinity treatments. Specifically, MDA levels rose significantly after one and two hours of storage delay, compared to the expected baseline following water loss. Under lower salinity conditions, the VM, Shik, and Meg ecotypes exhibited elevated MDA levels after one hour, whereas the Ruh and AM ecotypes showed values consistent with the anticipated outcome.

At higher salinity levels, all ecotypes exhibited significantly higher MDA concentrations than expected after dehydration. After two hours, both salinity treatments led to the highest MDA accumulation across the ecotypes, indicating increased oxidative stress due to prolonged water loss. The only exception was the Ruh ecotype, which maintained MDA levels similar to the anticipated baseline after water loss (**Figure 9**; **Supplementary Figures 3A, B**).

The one- and two-hour results were further validated by demonstrating that a two-hour delay before placing samples in cold room storage for 21 days yielded the highest MDA levels compared with the immediately stored control plants. Among the ecotypes, VM and Meg showed the largest increases in MDA following storage delay, whereas the Shik ecotype showed comparatively lower MDA accumulation (see **Figures 9**, **12**; **Supplementary Figures 3A, B**).

An increase in MDA indicates tissue damage and reduced vegetable quality, with elevated levels of the toxic aldehyde suggesting poor quality. Yet, while MDA levels in lettuce rose significantly during extended storage, from 35 nmol/g FW in the control group to 180 nmol/g FW after 15 days (Li et al., 2021), both the initial and final levels were substantially higher than those observed in *Sarcocornia fruticosa* ecotypes and AM vegetables, both before and after storage delay (**Figures 9**, **12**; **Supplementary Figures 3A, B**). Significantly, MDA levels in additional unstressed vegetables, such as pak choi, cucumber, flowering Chinese cabbage, and tomato (Ahammed et al., 2012), were several-fold higher than those reported in the current study. These results indicate that the increases in MDA observed during storage in the current study do not significantly impact the nutritional quality or safety of *Sarcocornia fruticosa* ecotypes and AM vegetables for human consumption.

### 4.3 One- and two-hour storage delays enhanced total polyphenol content in most harvested *Sarcocornia* ecotypes but not in *Arthrocaulon macrostachyum*

Polyphenols play a crucial role in plants’ resistance to abiotic stress by enhancing antioxidant capacity, protecting cellular membranes, and minimizing oxidative damage. Key classes of polyphenolic compounds, such as flavonoids, anthocyanins, and carotenoids, work together to scavenge ROS, stabilize membranes, and maintain cellular integrity under stressful conditions (Rao and Zheng, 2025; Sahu et al., 2022). The current study revealed differing responses in total polyphenol content to short postharvest storage delays between *Sarcocornia* ecotypes and AM. Total polyphenol content significantly increased after one- and two-hour storage delays across most ecotypes and salinity treatments, compared with the control samples, except in Ruh after two hours at higher salinity and in AM at both salinity levels and both storage delay durations (**Figure 13A, B**, **4**). In the *Sarcocornia* ecotypes, increases in polyphenol levels often exceeded those expected from water loss alone, indicating that active metabolic regulation is at play and demonstrating that short-term postharvest stress can quickly boost antioxidant metabolism in certain halophytic genotypes. However, the intensity and direction of this response varied among ecotypes and were influenced by salinity, underscoring the significance of genetic background in postharvest stress tolerance. In contrast, the AM ecotype consistently exhibited lower polyphenol levels under both salinity treatments, even after adjusting for water loss (**Figure 4**, **13A, B**).

**Figure 13:**
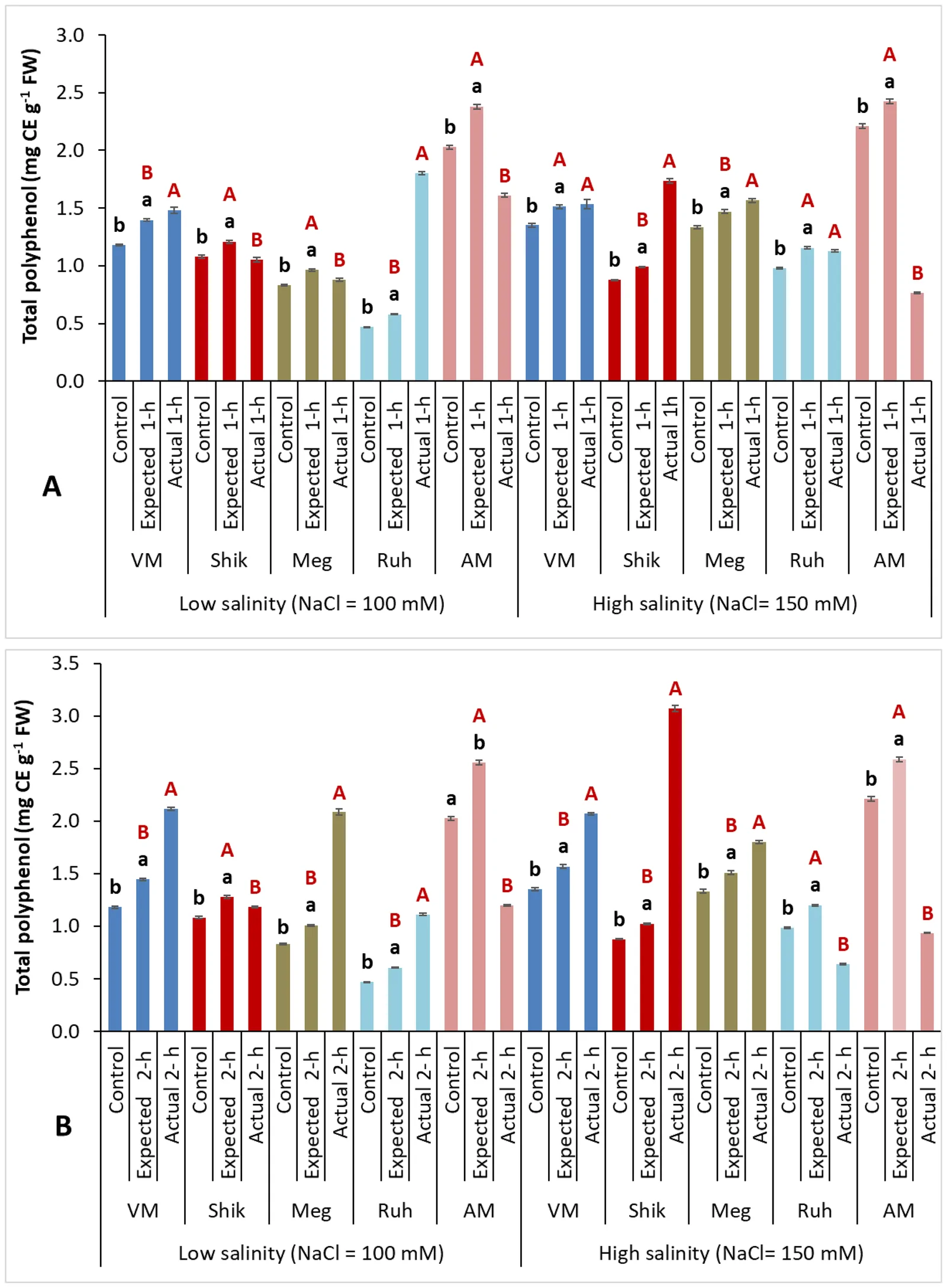
The calculated expected polyphenol level (considering the water loss compared to the control), compared with the actual level after one hour **(A)** and two hours **(B)** of storage delay in harvested shoots of *Sarcocornia* (VM, Shikmona [Shik], Megadim [Meg], and Ruhama [Ruh]), and *Arthrocaulon macrostachyum* (AM), irrigated with water containing 100 or 150 mM NaCl. The means plus standard errors (± SE) are shown. Significant differences between the expected and actual levels in a specific ecotype are indicated by different uppercase red letters, while differences between the control and the expected result in the same ecotypes are presented by different lowercase black letters under the same salinity treatment (Tukey’s HSD, p ≤ 0.05, n = 6).

Under the lower salinity treatment (100 mM), a one-hour storage delay resulted in significantly higher total polyphenol levels in the VM and Ruh ecotypes (exceeding the expected values by 5.71% and 210.34%, respectively). In contrast, Shik (13.22), Meg (8.33%), and AM (32.23%) showed lower-than-expected polyphenol concentrations (**Figure 13A**).

After a two-hour delay, VM, Meg, and Ruh exhibited notable increases above expected levels (47.22%, 108.07%, and 85%, respectively). In contrast, while the Shik ecotype showed higher values than the control plant, both Shik (7.81%) and AM (53.12%) showed reductions relative to anticipated water-loss-adjusted values (**Figure 13B**).

Under the 150 mM salinity treatment, after a one-hour storage delay, Shik and Meg exhibited total polyphenol concentrations that were significantly higher than expected (75.75% and 6.80%, respectively). In contrast, VM and Ruh remained close to their expected levels, while the AM (68.82%) ecotype showed a significant decline. After a two-hour delay, VM, Shik, and Meg demonstrated substantial increases in polyphenol content, surpassing the expected values (by 31.84%, 94.26%, and 192.3%, respectively). In contrast, Ruh (46.66%) and AM (63.7%) showed significant decreases relative to their anticipated water-loss-adjusted levels (**Figures 13A, B**).

These results indicate that the AM is particularly sensitive to unfavorable postharvest conditions arising from storage delays. Its reduced accumulation of polyphenols suggests a lower ability to activate antioxidant defenses than in the immediately stored harvested shoots.

### 4.4 One- and two-hour storage delays enhanced the radical scavenging activity in most *Sarcocornia* ecotypes but not in *Arthrocaulon macrostachyum*

Radical scavenging activity (RSA) reflects the ability of antioxidant compounds to neutralize free radicals and limit oxidative damage (Harboub et al., 2025). In this study, RSA was assessed using the DPPH (2,2-diphenyl-1-picrylhydrazyl) assay, a widely applied method for evaluating antioxidant potential (Choudhary et al., 2023). The current study identified distinct differences in radical scavenging responses to short postharvest storage delays between *Sarcocornia* ecotypes and AM. In *Sarcocornia* ecotypes, radical-scavenging activity generally increased after one- and/or two-hour storage delays compared to controls, regardless of salinity, indicating rapid activation of antioxidant capacity by short-term drought stress. In contrast, the AM consistently showed lower radical-scavenging activity after storage delays, suggesting a decrease in the antioxidant response to the stress (**Figure 8**).

The radical scavenging activity significantly increased in most *Sarcocornia* ecotypes after one and two hours of water loss during storage delay, compared to levels expected after water loss alone. At the lower salinity level, after one hour of storage delay, the radical scavenging activity increased above the expected level due to water loss by 28.18% in VM, 27.17% in Shik, 27.45% in Meg, and 24.6% in Ruh. After a two-hour delay, the increases were more pronounced for the *Sarcocornia* ecotypes, with VM increasing by 57.88%, Shik by 63.31%, and Ruh by 59.19%, while Meg showed results similar to those expected (**Figures 8**, **14A, B**).

**Figure 14:**
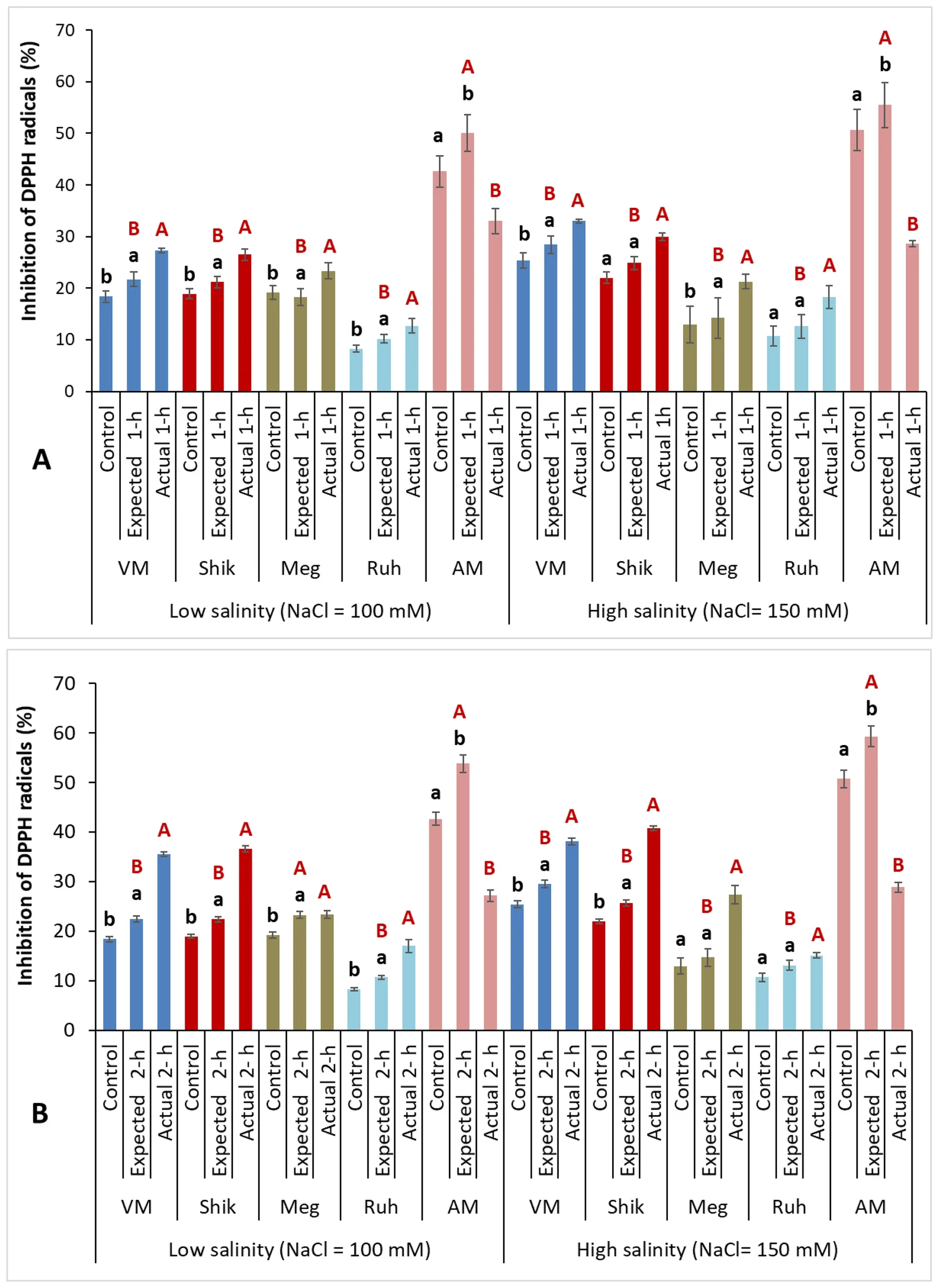
The calculated expected radical scavenging activities by DPPH level (considering the water loss compared to the control), compared with the actual after one hour **(A)** and two hours **(B)** of storage delay in harvested shoots of *Sarcocornia* (VM, Shikmona [Shik], Megadim [Meg], and Ruhama [Ruh]), and *Arthrocaulon macrostachyum* (AM), irrigated with water containing 100 or 150 mM NaCl. The means plus standard errors (± SE) are shown. Significant differences between the expected and actual levels in a specific ecotype are indicated by different uppercase red letters, while differences between the control and the expected result in the same ecotypes are presented by different lowercase black letters under the same salinity treatment (Tukey’s HSD, p ≤ 0.05, n = 5).

In the higher-salinity treatments, a similar pattern was observed after one and two hours of storage delay. The results showed increases in radical scavenging activity in VM by 16.1%, in Shik by 20.4%, in Meg by 49.29%, and in Ruh by 45.23% after a one-hour storage delay. After a 2-hour storage delay, the radical scavenging activity increased more than the expected level in VM by 29.06%, in Shik by 58.8%, and in Meg by 87.09%, and Ruh significantly increased by 15.46% (**Figures 8**, **14A, B**).

Overall, similar to the polyphenol content (**Figures 4**, **13A, B**), the AM ecotype demonstrated a significant decrease in radical scavenging activity compared to the expected response following water loss, in lower salinity by 31.66% after one hour and after two hours by 49.55%: in higher salinity decreased by 48.35% after one hour and after two hours by 51.35% (**Figures 8**, **14A, B**). This considerable reduction suggests that AM should be stored immediately, as it cannot maintain its high antioxidant capacity during such delays. Yet, although AM’s antioxidant capacity decreased when subjected to the two-hour storage delay treatment followed by 21 days of cold room storage, it remained higher than that of the *Sarcocornia* ecotypes across both types of antioxidant activities under both the immediately stored control and the two-hour storage delay treatments (**Figures 11B–D**).

### 4.5 Multivariate correlation between water loss, MDA, and antioxidant components

In the *Sarcocornia fruticosa* ecotypes, a principal component analysis (PCA) with Varimax rotation revealed two components that together accounted for 76.04% of the total variance. The first component (F1) explained 42.91% of the variance, while the second component (F2) accounted for 33.13%.

The positive F1 was primarily associated with antioxidant activity and oxidative stress-related traits, including malondialdehyde [MDA (R^2^ = 0.813)], radical scavenging activity (R^2^ = 0.879), total flavonoid content (R^2^ = 0.640), total polyphenol content (R^2^ = 0.537), carotenoids (R^2^ = 0.837), total soluble sugars (TSS) (R^2^ = 0.899), and electrical conductivity [EC (R^2^ = 0.868) (**Figure 15A**). The increases in TSS and EC reflect osmotic adjustment in response to water loss caused by storage delays. Similarly, increased MDA, reflecting lipid peroxidation, is likely related to the activation of the antioxidant components. The positive F2 was mainly characterized by anthocyanins (R^2^ = 0.935), indicating the drought-stress-induced accumulation of secondary metabolites and enhanced membrane protection by antioxidant components (**Figure 15A**).

**Figure 15:**
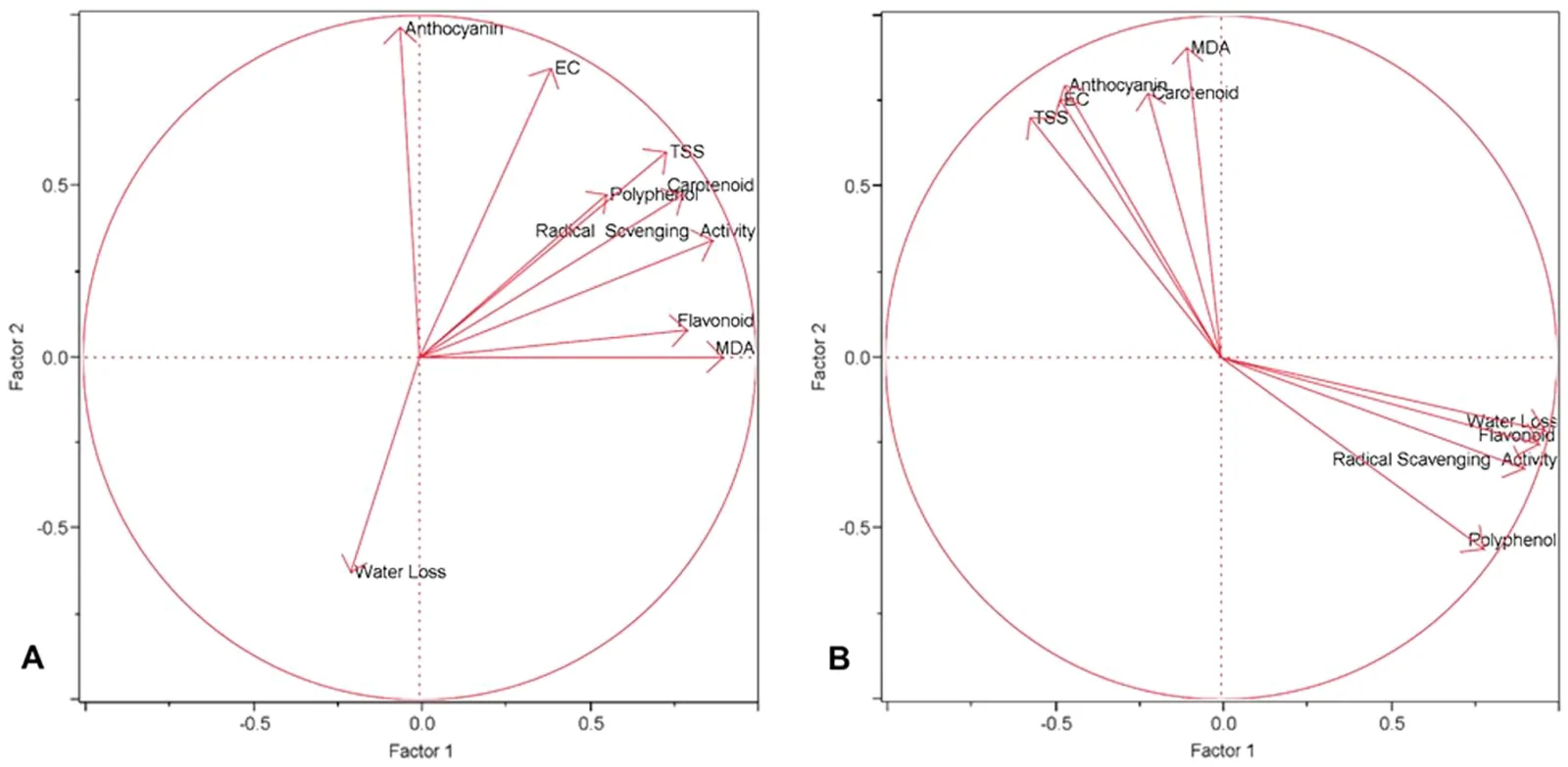
Principal component analysis (PCA) biplots depicting the relationships between postharvest physiological and biochemical traits in harvested *Sarcocornia fruticosa* ecotypes **(A)** and the *Arthrocaulon macrostachyum* ecotype **(B)**. The biplots demonstrate the correlations between these variables both under control conditions and after one- and two-hour postharvest storage delays in greenhouse conditions. The analysis used a multivariate approach with Varimax rotation and included the following variables: water loss, malondialdehyde (MDA), anthocyanins, electrical conductivity (EC), total soluble sugars (TSS), total carotenoids (carotenoids), total flavonoids (flavonoids), total polyphenols (polyphenols), and radical scavenging activity.

The water loss negative correlation [Negative F2 (R^2^ = 0.436)] demonstrates that as tissue water content decreases, the concentrations of osmotic component protectors such as TSS and EC, damage indicators such as MDA, and antioxidant components such as polyphenols, flavonoids, carotenoids, and anthocyanins increase to provide tissue protection, as indicated by radical scavenging activity (**Figure 15A**).

In the *Arthrocaulon macrostachyum* (AM) ecotype, principal component analysis (PCA) of nine physiological and biochemical traits showed that the first two principal components explained 76.04% of the total variability in the dataset. Principal component 1 (F1) accounted for 42.91% of the total variance (eigenvalue = 3.86), whereas principal component 2 (F2) explained 33.13% of the total variance (eigenvalue = 2.98). Detailed PCA results, including eigenvalues, percentage of variance explained, and factor loadings, are provided in **Supplementary Table 1**. Together, these two components captured the majority of the variation among the evaluated traits, indicating that they effectively summarize the physiological and biochemical responses of the AM ecotype. Detailed PCA results, including eigenvalues, percentage variance explained, and factor loadings, are presented in **Supplementary Table S1**. The positive F1 was strongly influenced by total flavonoids (R^2^ = 0.964), total polyphenols (R^2^ = 0.922), radical scavenging activity (R^2^ = 0.918), and water loss (R^2^ = 0.980) (**Figure 15B**). These results indicate that water loss is commonly associated with reductions in antioxidant capacity in AM.

In contrast, the positive F2 showed strong correlations among MDA (R^2^ = 0.832), EC (R^2^ = 0.801), anthocyanins (R^2^ = 0.852), carotenoids (R^2^ = 0.647), and total soluble sugar (R^2^ = 0.814) (**Figure 15B**), indicating significant biochemical responses. As water loss increases, antioxidant capacity declines, particularly affecting polyphenols, flavonoids, and radical-scavenging activity. This decline exacerbates oxidative damage, as evidenced by elevated MDA levels resulting from lipid peroxidation. At the same time, the increase in anthocyanins and carotenoids, along with total soluble sugar and EC, likely reflects adaptive or compensatory responses to stress.

The contrasting correlations between water loss and antioxidant traits indicate species-specific postharvest adaptation strategies. In *Sarcocornia fruticosa*, moderate water loss during a one- or two-hour pre-cold-storage delay promoted the accumulation of antioxidant compounds (polyphenols, flavonoids, carotenoids, and anthocyanins) and enhanced radical-scavenging activity, suggesting that mild dehydration acts as a beneficial stress signal that improves nutritional quality without causing substantial oxidative damage. Therefore, a short, controlled delay before cold storage may enhance the nutritional value of *S. fruticosa* ecotypes.

In contrast, *Arthrocaulon macrostachyum* showed weak or negative associations between water loss and antioxidant metabolites, accompanied by increased oxidative stress, indicating limited tolerance to postharvest dehydration. Consequently, immediate cooling after harvest is recommended to minimize water loss and preserve its nutritional quality and visual appearance. Overall, the PCA highlights species-specific postharvest responses: Short storage delays may stimulate antioxidant accumulation in *S. fruticosa* ecotypes, whereas minimizing dehydration and applying rapid cooling are more appropriate for AM species to preserve biochemical stability and marketable quality. Further studies are needed to identify the mechanisms that affect the nutritional quality of *Sarcocornia* ecotypes and *Arthrocaulon macrostachyum* ecotype plant material after harvest during storage delays and cold storage.

## 5 Conclusion

The effects of one- and two-hour postharvest storage delays on the nutritional quality of halophytic plants irrigated with 100 or 150 mM NaCl were assessed. The study compared the new *Sarcocornia fruticosa* ecotypes Shikmona, Megadim, and Ruhama with the commonly cultivated VM and *Arthrocaulon macrostachyum.* Unlike traditional leafy vegetables, harvested *Sarcocornia fruticosa* demonstrated enhanced antioxidant and nutritional properties following a short storage delay. Delaying storage in *Sarcocornia* by one or two hours significantly enhanced the total polyphenols, flavonoids, carotenoids, anthocyanins, and radical scavenging activity, compared with the immediately stored control. This enhancement in the storage-delay samples was also observed after storing the control and two-hour storage-delay samples for 21 days. Notably, the new *Sarcocornia* ecotypes, Shikmona (Shik), Megadim (Meg), and Ruhama (Ruh), outperformed the locally used VM cultivar in both nutritional composition and yield.

Furthermore, the one and two-hour storage delays significantly increased the antioxidant capacity of the *Sarcocornia* shoot product beyond the expected increase due to water loss. In contrast, *Arthrocaulon macrostachyum* exhibited a decrease in antioxidant levels, except for total anthocyanins and carotenoids. Both storage delays increased total soluble sugar, EC, and malondialdehyde levels in *Sarcocornia* ecotypes and in *Arthrocaulon macrostachyum*. Further studies are needed to identify the mechanisms that affect the nutritional quality of *Sarcocornia* ecotypes and *Arthrocaulon macrostachyum* ecotype plant material after harvest during storage delays and cold storage.

## Data Availability

The original contributions presented in the study are included in the article/**Supplementary Material**. Further inquiries can be directed to the corresponding author.
