# Supplementary material for: One- and two-hour storage delays improve nutritional components in Sarcocornia fruticosa ecotypes, but not in the Arthrocaulon macrostachyum ecotype
Source: Front Plant Sci. 2026 Oct 6;17:1943035. doi: 10.3389/fpls.2026.1943035 (PMC13681455; doi:10.3389/fpls.2026.1943035)
Supplement: Supplementary file 1 [file Supplementaryfile1.docx]

**Supplementary Material Figures**

**1. Total soluble sugar contents**

**Supplementary Figure 1**. The calculated expected total soluble sugar (considering the water loss compared to the control), compared with the actual level after one hour (A) and two hours (B) of storage delay in harvested shoots of *Sarcocornia* (VM, Shikmona [Shik], Megadim [Meg], and Ruhama [Ruh]), and *Arthrocaulon macrostachyum* (AM), irrigated with water containing 100 or 150 mM NaCl. The means plus standard errors (±SE) are shown. Significant differences between the expected and actual levels in a specific ecotype are indicated by different uppercase red letters, while differences between the control and the expected result in the same ecotypes are presented by different lowercase black letters under the same salinity treatment ('Tukey's HSD, p ≤ 0.05, n = 3).

**2. Electrical conductivity levels**

**Supplementary** **Figure 2.** The calculated expected electrical conductivity level (considering the water loss compared to the control), compared with the actual level after one hour (A) and two hours (B) of storage delay in harvested shoots of *Sarcocornia* (VM, Shikmona [Shik], Megadim [Meg], and Ruhama [Ruh]), and *Arthrocaulon macrostachyum* (AM), irrigated with water containing 100 or 150 mM NaCl. The means plus standard errors (±SE) are shown. Significant differences between the expected and actual levels in a specific ecotype are indicated by different uppercase red letters, while differences between the control and the expected result in the same ecotypes are presented by different lowercase black letters under the same salinity treatment (Tukey's HSD, p ≤ 0.05, n = 3).

**3. Malondialdehyde (MDA) levels**

**Supplementary Figure 3.** The calculated expected malondialdehyde level (considering the water loss compared to the control), compared with the actual level after one hour (A) and two hours (B) of storage delay in harvested shoots of *Sarcocornia* (VM, Shikmona [Shik], Megadim [Meg], and Ruhama [Ruh]), and *Arthrocaulon macrostachyum* (AM), irrigated with water containing 100 or 150 mM NaCl. The means plus standard errors (±SE) are shown. Significant differences between the expected and actual levels in a specific ecotype are indicated by different uppercase red letters, while differences between the control and the expected result in the same ecotypes are presented by different lowercase black letters under the same salinity treatment (Tukey's HSD, p ≤ 0.05, n = 3).

**Supplementary Figure 4**. **Experimental Design: Schematic Representation of Sampling and Storage Treatments**

**Supplementary Figure 5**. **Integrated schematic model depicting the variation in growth, biochemical traits, and stress-responsive behavior of different ecotypes.**

**Supplementary Figure 5. Integrated schematic model illustrating the responses of different ecotypes under varying salinity and postharvest delay treatments.** The symbols **lower (****), moderate (****),** and **higher (** **)** indicate significant differences among postharvest treatments (control, 1 h, and 2 h) within the same ecotype and salinity level. The symbols **decreased (****)** and **increased (****)** represent significant differences between salinity treatments within the same ecotype and corresponding postharvest treatment (i.e., comparisons between control vs. control, 1 h vs. 1 h, and 2 h vs. 2 h storage delays). The schematic summarizes the relative changes in physiological and biochemical responses across ecotypes in relation to salinity exposure and postharvest storage duration.

**Supplementary Table 1 for *Arthrocaulon macrostachyum***

| **Trait** | **F1 loading** | **F2 loading** | **R² (Communality)** |
| --- | --- | --- | --- |
| Total flavonoid | 0.948 | -0.256 | 0.964 |
| Total polyphenol | 0.781 | -0.559 | 0.922 |
| Radical scavenging activity (RSA) | 0.901 | -0.326 | 0.918 |
| Water loss | 0.966 | -0.214 | 0.98 |
| Total carotenoid | -0.216 | 0.775 | 0.647 |
| MDA | -0.102 | 0.906 | 0.832 |
| TSS | -0.567 | 0.702 | 0.815 |
| EC | -0.479 | 0.756 | 0.801 |
| Anthocyanin | -0.468 | 0.796 | 0.852 |

**Supplementary Table 2. Factorial Multi-Way ANOVA (Ecotype × Salinity × Storage Delay)**

| **Assay** | **Source** | **DF** | **Sum of Squares** | **F -Value** | **P-Value** |
| --- | --- | --- | --- | --- | --- |
| Total carotenoid | Ecotypes | 4 | 6453.38 | 1110.39 | <.0001* |
|  | Storages | 2 | 3157.89 | 1086.71 | <.0001* |
|  | Salinity | 1 | 882.21 | 607.18 | <.0001* |
|  | Ecotypes*Storages | 8 | 864.99 | 74.42 | <.0001* |
|  | Storages*Salinity | 2 | 285.81 | 98.35 | <.0001* |
|  | Ecotypes*Salinity | 4 | 57.21 | 9.84 | <.0001* |
|  | Ecotypes*Storages*Salinity | 8 | 569.95 | 49.03 | <.0001* |
|  | Error | 150 | 217.944 |  |  |
| Anthocyanin | Ecotypes | 4 | 0.08 | 1048.37 | <.0001* |
|  | Storages | 2 | 0.03 | 713.70 | <.0001* |
|  | Salinity | 1 | 0.01 | 494.03 | <.0001* |
|  | Ecotypes*Storage | 8 | 0.00 | 5.12 | <.0001* |
|  | Storages*Salinity | 2 | 0.00 | 14.22 | <.0001* |
|  | Ecotypes*Salinity | 4 | 0.01 | 111.90 | <.0001* |
|  | Ecotypes*Storages*Salinity | 8 | 0.00 | 8.87 | <.0001* |
|  | Error | 60 | 0.001104 |  |  |
| Total flavonoid | Ecotypes | 4 | 8.41 | 1249.23 | <.0001* |
|  | Storages | 2 | 0.40 | 119.01 | <.0001* |
|  | Salinity | 1 | 0.16 | 92.26 | <.0001* |
|  | Ecotypes*Storages | 8 | 9.66 | 717.12 | <.0001* |
|  | Storages*Salinity | 2 | 0.25 | 74.62 | <.0001* |
|  | Ecotypes*Salinity | 4 | 0.33 | 49.21 | <.0001* |
|  | Ecotypes*Storages*Salinity | 8 | 0.63 | 46.61 | <.0001* |
|  | Error | 150 | 0.252463 |  |  |
| Total polyphenol | Ecotypes | 4 | 7.42 | 1137.29 | <.0001* |
|  | Storages | 2 | 6.56 | 2010.91 | <.0001* |
|  | Salinity | 1 | 0.73 | 445.64 | <.0001* |
|  | Ecotypes*Storages | 8 | 20.67 | 1584.91 | <.0001* |
|  | Storages*Salinity | 2 | 1.33 | 408.94 | <.0001* |
|  | Ecotypes*Salinity | 4 | 7.00 | 1073.60 | <.0001* |
|  | Ecotypes*Storages*Salinity | 8 | 13.00 | 996.58 | <.0001* |
|  | Error | 150 | 0.244509 |  |  |
| Radical scavenging activity | Ecotypes | 4 | 8673.25 | 560.24 | <.0001* |
|  | Storages | 2 | 949.56 | 122.67 | <.0001* |
|  | Salinity | 1 | 246.87 | 63.78 | <.0001* |
|  | Ecotypes*Storages | 8 | 4558.22 | 147.22 | <.0001* |
|  | Storage*Salinity | 2 | 3.77 | 0.49 | 0.616 |
|  | Ecotypes*Salinity | 4 | 102.90 | 6.65 | <.0001* |
|  | Ecotypes*Storages*Salinity | 8 | 444.72 | 14.36 | <.0001* |
|  | Error | 120 | 464.438 |  |  |
| Total soluble sugar (TSS) | Ecotype | 4 | 88.96 | 87.02 | <.0001* |
|  | Storages | 2 | 144.82 | 283.35 | <.0001* |
|  | Salinity | 1 | 45.51 | 178.09 | <.0001* |
|  | Ecotype*Storages | 8 | 2.18 | 1.07 | 0.3994 |
|  | Storages*Salinity | 2 | 2.02 | 3.96 | 0.0243* |
|  | Ecotype*Salinity | 4 | 2.82 | 2.76 | 0.0356* |
|  | Ecotype*Storages*Salinity | 8 | 0.98 | 0.48 | 0.8668 |
|  | Error | 60 | 15.33333 |  |  |
| Electrical conductivity (EC) | Ecotypes | 4 | 2056.71 | 60.05 | <.0001* |
|  | Storages | 2 | 4749.09 | 277.32 | <.0001* |
|  | Salinity | 1 | 2030.65 | 237.16 | <.0001* |
|  | Ecotypes*Storages | 8 | 79.73 | 1.16 | 0.3357 |
|  | Storages*Salinity | 2 | 25.26 | 1.47 | 0.237 |
|  | Ecotypes*Salinity | 4 | 263.71 | 7.70 | <.0001* |
|  | Ecotypes*Storages*Salinity | 8 | 331.45 | 4.84 | 0.0001* |
|  | Error | 60 | 513.745 |  |  |
| Malondialdehyde (MDA) | Ecotypes | 4 | 761.58 | 1389.71 | <.0001* |
|  | Storages | 2 | 521.93 | 1904.83 | <.0001* |
|  | Salinity | 1 | 155.67 | 1136.24 | <.0001* |
|  | Ecotypes*Storages | 8 | 111.72 | 101.93 | <.0001* |
|  | Storages*Salinity | 2 | 122.43 | 446.83 | <.0001* |
|  | Ecotypes*Salinity | 4 | 41.95 | 76.55 | <.0001* |
|  | Ecotypes*Storages*Salinity | 8 | 46.01 | 41.98 | <.0001* |
|  | Error | 90 | 12.3302 |  |  |

**Supplementary Table 3. Experimental actual treatment arrangement (layout)**

| **Ecotype** | **Salinity** | **0 h Control** | **1 h** | **2 h** |
| --- | --- | --- | --- | --- |
| VM | 100 mM | ✓ | ✓ | ✓ |
| VM | 150 mM | ✓ | ✓ | ✓ |
| Shikmona | 100 mM | ✓ | ✓ | ✓ |
| Shikmona | 150 mM | ✓ | ✓ | ✓ |
| Megadim | 100 mM | ✓ | ✓ | ✓ |
| Megadim | 150 mM | ✓ | ✓ | ✓ |
| Na'aman | 100 mM | ✓ | ✓ | ✓ |
| Na'aman | 150 mM | ✓ | ✓ | ✓ |
| Ruhama | 100 mM | ✓ | ✓ | ✓ |
| Ruhama | 150 mM | ✓ | ✓ | ✓ |
| *Arthrocaulon macrostachyum* | 100 mM | ✓ | ✓ | ✓ |
| *Arthrocaulon macrostachyum* | 150 mM | ✓ | ✓ | ✓ |
